# Vibronic coupling-driven symmetry breaking and solvation in the photoexcited dynamics of quadrupolar dyes

**DOI:** 10.1038/s41557-025-01908-7

**Published:** 2025-08-20

**Authors:** Katrin Winte, Somayeh Souri, Daniel C. Lünemann, Fulu Zheng, Mohamed El-Amine Madjet, Thomas Frauenheim, Teresa Kraus, Elena Mena-Osteritz, Peter Bäuerle, Sergei Tretiak, Antonietta De Sio, Christoph Lienau

**Affiliations:** 1 Institut für Physik, Carl von Ossietzky Universität, Oldenburg, Germany; 2 Bremen Center for Computational Materials Science, Universität Bremen, Bremen, Germany; 3 School of Science, Constructor University, Bremen, Germany; 4 School of Physical Science and Technology, Ningbo University, Ningbo, China; 5 Institute for Advanced Study, Chengdu University, Chengdu, China; 6 Institut für Organische Chemie II und Neue Materialien, Universität Ulm, Ulm, Germany; 7 Theoretical Division and Center for Integrated Nanotechnologies, Los Alamos National Laboratory, Los Alamos, NM USA; 8 Center for Nanoscale Dynamics (CeNaD), Carl von Ossietzky Universität, Oldenburg, Germany

**Keywords:** Excited states, Nanoscale materials

## Abstract

Quadrupolar dyes, such as acceptor–donor–acceptor molecules, are highly relevant for applications in nonlinear optics and photovoltaics. They are also versatile models for exploring photoinduced charge-transfer dynamics. The interplay between electronic and vibronic couplings in these molecules may break excited-state symmetry, resulting in intramolecular charge separation and pronounced solvatochromism. Experimentally, distinguishing the roles of intramolecular vibronic coupling and solvent reorganization for the initial charge-transfer dynamics has been challenging so far. Here we investigate a prototypical quadrupolar dye in polar and non-polar solvents using ultrafast pump–probe and two-dimensional electronic spectroscopy. Our results reveal that vibronic couplings initiate excited-state symmetry breaking during the first ~50 fs of the photoinduced charge transfer, whereas solvent-induced charge localization sets in at later times. Quantum dynamics and electronic structure simulations support our experimental findings. Our results reveal the details of solvation dynamics in photoexcited molecules and suggest strategies for their manipulation through vibronic couplings.

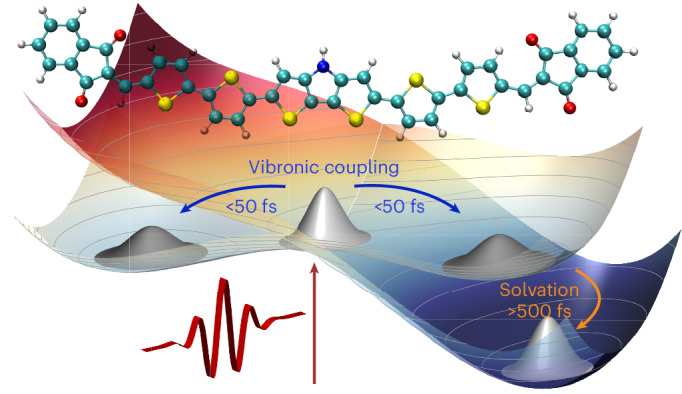

## Abstract (web summary)

Photoexcitation of quadrupolar dyes—key materials for various optoelectronic applications—induces an excited-state symmetry-breaking charge-transfer process with unknown microscopic origin. Now it has been shown that vibronic coupling to high-frequency backbone modes drives the initial ultrafast symmetry breaking before solvation, distinguishing fundamental intramolecular dynamics from solvent-induced charge localization.

## Main

Solvation is a fundamental process in chemistry, biology and physics. It is defined as a dynamical interaction of a molecular system, the solute, with its dielectric environment, the solvent^1,2^. It can profoundly influence charge-transfer reactions^2–5^, protein folding^6^, and the dynamics of electrons^7,8^ and ions^9^ in liquids. Although solvation has fascinated scientists for more than a century^10^, and its timescales are relatively well known^1,2,11^, fully unraveling the interplay between intramolecular dynamics and solvent-driven reorganization remains challenging. This is particularly true for highly non-equilibrium dynamics in electronically excited states initiated by optical excitation. Here, strong coupling between electronic and vibrational degrees of freedom, universal in molecules^12,13^, initiates ultrafast charge and energy redistributions within the molecule, and relaxation toward a new equilibrium state. The impact of the solvent environment on the relaxation dynamics is particularly pronounced when the molecular non-equilibrium charge distribution differs substantially from that in the ground state, inducing a solvent rearrangement in response to the charge displacement upon photoexcitation^1^. The signature of the solvent-induced relaxation is commonly observed as solvatochromism, that is, a red-shift between emission and absorption of the molecule that depends on solvent polarity.

**Fig. 1:**
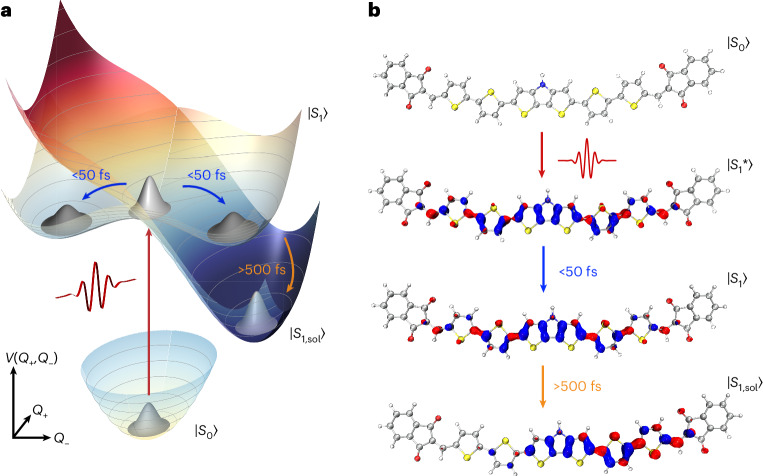
Conceptual illustration of polar solvation dynamics of a quadrupolar A–D–A molecule. **a**, Schematic potential energy surface V(*Q*_+_,*Q*_−_). **b**, Ball-and-stick representation of the investigated A–D–A molecule, which comprises a central dithienopyrrole–thiophene donor unit linked to two symmetric indandione acceptor groups. Snapshots of the local excess electron (red) and hole (blue) densities in the relevant excited states are overlaid. The orbital plots (isovalue = 0.002) are obtained from quantum-chemical calculations accounting for state-specific solvation (see Methods) being critical to model excited-state symmetry-breaking caused by the polar solvent. Resonant photoexcitation brings the molecule from the neutral ground state $\left|{S}_{0}\right\rangle$ to a non-equilibrium region $\left|{S}_{1}^{* }\right\rangle$ of the excited state with a symmetric charge distribution that is delocalized across the molecule. Vibronic couplings to symmetric (*Q*_+_) and antisymmetric (*Q*_−_) combinations of high-frequency backbone vibrations on each arm of the molecule result in a symmetry-broken double-minimum $\left|{S}_{1}\right\rangle$ surface along *Q*_−_. The interaction with a polar solvent environment tilts the $\left|{S}_{1}\right\rangle$ potential along *Q*_−_, lowering the energy of one of the minima. This induces relaxation towards the solvated excited-state minimum $\left|{S}_{1,{\rm{sol}}}\right\rangle$ on a slower timescale and, concurrently, localizes the charges preferentially on one arm of the molecule.

Photoinduced relaxation dynamics in molecules typically involve an ultrafast electronic response of the dielectric environment, followed by a slower solvent rearrangement and equilibration (Fig. 1a). The interplay between intramolecular electron–vibrational (that is, vibronic) dynamics and solvent rearrangement may lead to spatial localization of the photoexcitation (Fig. 1a). These processes frequently occur in a non-adiabatic regime beyond the Born–Oppenheimer approximation^12,13^. The new metastable excited state then persists until radiative or non-radiative decay brings the molecule back into its ground state. The initial non-adiabatic dynamics often occur on timescales governed by high-frequency intramolecular vibrations, such as carbon–carbon (C–C) stretching motions with ~20 fs periods, thus challenging to address experimentally. Also, first-principles simulation of excited-state non-adiabatic dynamics and solvation is a highly demanding computational task^13^.

This interplay between vibronic couplings and rearrangement of the dielectric environment also underlies polaron physics in condensed phase systems that feature strong coupling of the electronic system to a polar lattice^14^. Polaronic excitations dominate the optical and transport properties of various functional materials^14^, including polar semiconductors^15^, molecular aggregates^16,17^, conjugated polymers^18–20^ and halide perovskites^21–24^. As polaron formation involves vibronic dynamics on ultrafast timescales of only few tens of femtoseconds, experimental studies directly probing these processes have emerged only recently^18,25–29^. As such, a detailed understanding of the dynamical interplay between vibronic couplings and solvation is currently of high interest for both molecular photophysics in solution and for light-induced processes in condensed matter.

Quadrupolar dyes represent a versatile, chemically tunable class of chromophores^30–33^ for exploring these photo-induced processes. They have recently attracted attention as materials for solution-processable photovoltaics^30,34,35^, sensing applications^36^ and for their interesting nonlinear and chiroptical properties^37,38^. They feature two terminal acceptor (donor) units linked to a central donor (acceptor) group, resulting in acceptor–donor–acceptor (A–D–A) or D–A–D structures. Electronic coupling between the two D–A arms induces wavefunction delocalization and its strength controls the charge-transfer character of the electronic states^39,40^. Furthermore, in each D–A arm, the electronic states are strongly coupled to high-frequency C–C stretching vibrations. In an intermediate coupling regime, comparable electronic and vibronic coupling strengths yield a highly anharmonic, double-minimum shape of the effective excited-state potential energy surface (PES)^39^ (Fig. 1a). In this case, the photoexcited charge density is initially symmetrically distributed across the entire molecular backbone. Excited-state solvation may induce a dynamical symmetry breaking, preferentially localizing the charge density on one arm^39,41–48^, resulting in solvatochromism. While solvation dynamics have been studied using various spectroscopic techniques^49,50^, the origin of symmetry breaking—whether initiated by intramolecular vibronic couplings or solvent reorientation—has not yet been revealed. By contrast, strong electronic coupling between the D–A arms, exceeding vibronic coupling in each arm, leads to robust charge delocalization across the molecular backbone, making the optical response largely insensitive to solvation. Squaraine dyes are prototypical examples of this behaviour^39^.

Symmetry-breaking charge transfer between identical chromophores has been explored in various chemically linked oligomers^51–54^, which mimic charge separation in photosynthetic reaction centres and serve as photoactive components in photovoltaics and photocatalysis. Recent work has investigated the effect of spatial separation and torsional rigidity for symmetry-breaking dynamics in a series of BODIPY dimers^51^, whereas, in perylene bisimide dimers, symmetry breaking is suggested to be activated by both structural and solvent fluctuations from an excimer state^53^. In a symmetric D–A–D triad, signatures for ultrafast sub-100-fs formation of charge-transfer states from a locally excited state on the acceptor moiety, along with the appearance of vibrational coherence of low-frequency vibrations in the charge-transfer state, have been recently reported^55^. These studies, however, do not provide insights into the potential role of high-frequency vibrations in the symmetry-breaking process.

Here we explore these dynamics in a (quasi-)quadrupolar A–D–A dye 2,2′-{{[4-(2-hexyldecyl)-4*H*-dithieno[3,2-*b:*2′,3′-*d*]pyrrol-2,6-diyl]bis[3,4′-dihexyl-(2,2′-bithiophene)-5′,4-diyl]}bis(methaneylylidene)}bis(1*H*-inden-1,3(2*H*)-dione) comprising two terminal indandione acceptors linked to a central dithienopyrrole–thiophene donor (Fig. 1b). Using ultrafast pump–probe and two-dimensional electronic spectroscopy (2DES) with sub-10-fs time-resolution, we probe the photoinduced charge-transfer dynamics of the A–D–A dye dissolved in non-polar and polar solvents. In both solvents, our results unveil an initial, ~50 fs timescale dictated by intramolecular vibronic couplings and largely unaffected by solvation. Only on a slower timescale we also observe solvent-induced relaxation. The salient quantum dynamics are well described by a reduced model Hamiltonian comprising the ground state and two excited electronic states coupled to two effective vibrational modes.

## Results

### Steady-state optical spectra

**Fig. 2:**
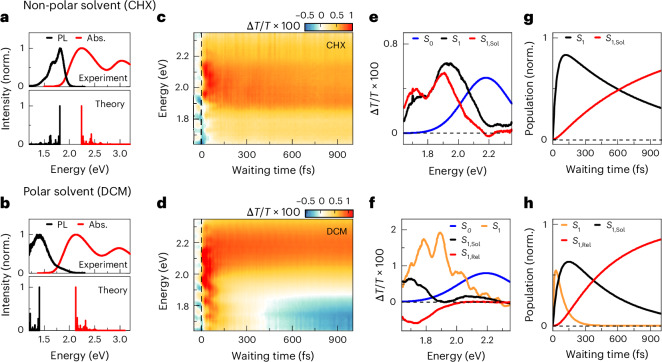
Ultrafast dynamics in non-polar and polar solvents. **a,b**, Experimental and simulated linear absorption (red) and photoluminescence (black) spectra of the A–D–A molecule dissolved in non-polar CHX (**a**) and polar DCM (**b**). **c,d**, Differential transmission Δ*T*/*T* maps of the molecule in CHX and DCM. A persistent ground-state bleaching band around 2.1 eV monitors $\left|{S}_{0}\right\rangle \to \left|{S}_{1}^{* }\right\rangle$ transitions. **c**, In CHX, a long-lived band below 2 eV probes stimulated emission from the relaxed excited state $\left|{S}_{1}\right\rangle \to \left|{S}_{0}\right\rangle$. **d**, In DCM, this stimulated emission transiently red-shifts and decays within ~400 fs ($\left|{S}_{1,{\rm{Sol}}}\right\rangle$) while, concurrently, absorption from the solvated excited state ($\left|{S}_{1,{\rm{Rel}}}\right\rangle$) builds up. In both solvents, the dynamics are modulated by persistent ~1,430 cm^−1^ C–C stretching oscillations. **e,f**, Incoherent spectral components of the Δ*T*/*T* in CHX (**e**) and DCM (**f**) deduced by a global analysis of the Δ*T*/*T* data in **c** and **d**, respectively. **g,h**, Population dynamics corresponding to the spectra in **e** and **f**, respectively. The dynamics are normalized to the total population of the excited state.

The linear spectra of the A–D–A molecule in the non-polar solvent cyclohexane (CHX) lack the typical mirror symmetry between absorption and emission (Fig. 2a). The absorption shows a broad, structureless transition from the ground state, $\left|{{\rm{S}}}_{0}\right\rangle$, to the Franck–Condon region of the lowest excited state, $\left|{{\rm{S}}}_{1}^{* }\right\rangle$, at ~2.1 eV (Fig. 2a, red, upper panel) due to torsional disorder present in the ground-state structures with pronounced bond-length alternation pattern^56^. The emission lineshape is considerably narrower and displays pronounced vibronic substructure with ~180 meV peak spacing. The torsions in the excited-state PES are reduced due to the quinoid structure and so the molecules become more planar^56^ (Supplementary Table 1). A weaker absorption at ~3 eV reflects transitions to higher excited states^28^. Although the absorption shows minimal solvent dependence, the emission substantially red-shifts in polar DCM (Fig. 2a,b). The Stokes shift increases considerably—from 430 meV in CHX to ~780 meV in DCM—while the emission lineshape broadens. Moreover, the emission quantum yield substantially decreases from ~34% in CHX to <1% in DCM. These observations indicate red-shifted and quenched emission from a polar excited state (Fig. 1a, $\left|{S}_{1,{Sol}}\right\rangle$), characteristic of solvation of a dipolar molecule. The spectral shape and pronounced Stokes shift in DCM are qualitatively reproduced by quantum-chemical calculations accounting for state-specific solvation (Fig. 2a,b). These indicate that the vibronic substructure is dominated by high-frequency C–C stretching modes of the conjugated backbone at ~1,500 cm^−1^ with a Huang–Rhys factor of ~0.28 (Supplementary Figs. 16–20). Quantum-chemical calculations show a non-vanishing dipolar component in the molecule making it, strictly speaking, quasi-quadrupolar (Supplementary Table 1).

### Ultrafast dynamics of the photoexcited quadrupolar dye

To analyse the ultrafast photoinduced dynamics, we record differential transmission Δ*T*/*T* spectra (Fig. 2c,d) of the A–D–A molecule in both solvents at room temperature. We excite and probe with broadband 8 fs pulses at around 2.1 eV (Supplementary Fig. 1). The duration of these pulses is much shorter than the period of the high-frequency modes. The neat solvent response has been carefully subtracted (see Methods).

In both solvents, optical excitation leads to a ground-state bleaching (GSB) band, $\Delta T/T > 0$, centred around 2.1 eV (Fig. 2c,d), which reflects pump-induced population transfer from $\left|{S}_{0}\right\rangle$ to $\left|{S}_{1}^{* }\right\rangle$. The associated spectrum resembles the absorption, and persists much beyond our 1 ps measurement window. The data in CHX reveal an additional stimulated emission (SE) band, $\Delta T/T > 0$, which is slightly red-shifted compared with the absorption. Here the probe induces population back-transfer from the excited state to the ground state. This SE persists across the entire waiting time range. In DCM, a related SE appears only during the first 300 fs, and exhibits a more pronounced red-shift than in CHX, extending beyond 1.6 eV, which is the lower limit of our probe energy range. This red-shifted SE unambiguously indicates polar solvation of the A–D–A molecule, probing the build-up of the pronounced solvatochromism observed in Fig. 2b (refs. ^39,43,57^). Beyond 300 fs, this SE is replaced by a long-lived excited-state absorption (ESA), $\Delta T/T < 0$, centred around 1.8 eV. We assume that this probes transitions from the solvated, vibrationally relaxed excited state $\left|{S}_{1,{\rm{Rel}}}\right\rangle$ to higher-lying states. In addition to those comparatively slow dynamics, the data show weak, persistent oscillations with a 23 fs period (1,430 cm^−1^), probably reflecting ground-state coherent vibrational wavepacket motion along the high-frequency backbone modes. At early waiting times <100 fs, faint coherent modulations of the Δ*T*/*T* data are observed in both solvents (Fig. 2c,d).

**Fig. 3:**
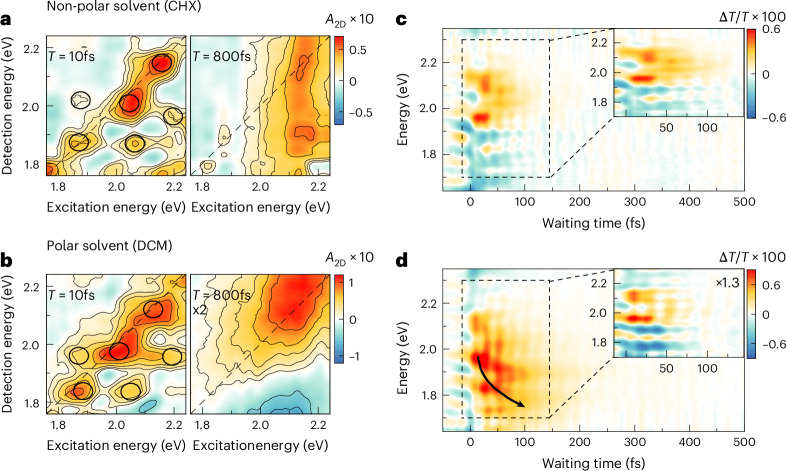
Vibronically driven intramolecular dynamics in quadrupolar dyes. **a,b**, Two-dimensional electronic spectroscopy maps of the A–D–A molecule in CHX (**a**) and DCM (**b**) at selected waiting times (*T*) of 10 fs and 800 fs. At early times, the 2DES maps reveal a pronounced vibronic peak structure (black circles) arising from vibrational wavepacket motion on the $\left|{S}_{1}\right\rangle$ PES dominated by the symmetric C–C stretching mode. The early time maps are very similar in both solvents. At later times, washout of the vibronic peaks leaves a broad stimulated emission peak from $\left|{S}_{1}\right\rangle$ in CHX, whereas in DCM excited-state absorption from $\left|{S}_{1,{\rm{Rel}}}\right\rangle$ builds up. **c,d**, Residual differential transmission Δ*T*/*T* maps at early times in CHX (**c**) and DCM (**d**) obtained by subtracting the long-lived components (blue, black and red in Fig. 2e–h) from the corresponding Δ*T*/*T* maps in Fig. 2c,d. **d**, In DCM, polar solvation gives rise to a red-shifting stimulated emission (**d**, black arrow) at energies below 2 eV, which is absent in CHX (**c**). The black arrow in **d** is a guide to the eyes. Further subtraction of the solvent-induced component (orange in Fig. 2f,h) from the DCM map uncovers vibronic lineshapes and sub-50 fs dynamics similar in both solvents (insets). This suggests that these initial dynamics are mainly governed by intramolecular vibronic couplings while the solvent polarity has minor effect.

Further details on the solvation dynamics are obtained through global analysis of the Δ*T*/*T* spectra (Supplementary Section 2). Decomposing Δ*T*/*T* into a series of exponential relaxation components with time-independent spectra faithfully models all incoherent contributions for waiting times >50 fs using a small number of decays with transparent physical meaning. The deduced GSB spectra (Fig. 2e,f, blue) reveal that ground-state refilling occurs well beyond our measurement timescale in both solvents. In CHX, the analysis uncovers two spectrally slightly shifted SE components (Fig. 2e, black and red). The high-energy band (black) decays with 850 fs, the build-up time of the low-energy band (red) (Fig. 2g). This 850 fs timescale is slower than the intramolecular vibrational relaxation time of <100 fs deduced from the decay of the excited-state wavepacket motion in Fig. 3. It may reflect slower cooling processes in the molecule by solvent reorganization dynamics and vibrational energy transfer to the solvent^58^. In DCM, the dominant SE is markedly red-shifted ($\left|{S}_{1,{\rm{Sol}}}\right\rangle$) (Fig. 2f, black) and vanishes on a faster timescale of 450 fs, whereas the ESA ($\left|{S}_{1,{\rm{Rel}}}\right\rangle$) (Fig. 2f, red) builds up. The 450 fs timescale matches well the solvent relaxation time in DCM^2,59^, supporting the assignment of the 1.7 eV band to ESA from $\left|{S}_{1,{\rm{Rel}}}\right\rangle$. Furthermore, analysis of the DCM data reveals a broad, vibronically structured SE band decaying within 80 fs (Fig. 2f,h, orange), again pointing to fast initial solvation steps.

We also employ 2DES with the same 8 fs pulses to analyse these early time solvation dynamics in greater detail. Figure 3a,b displays representative 2DES maps for CHX and DCM. Maps recorded at additional waiting times are reported in Supplementary Fig. 9. All 2DES data have been corrected for the neat solvent response, as for the Δ*T*/*T* data (see Methods and Supplementary Fig. 8). Initially (*T* = 10 fs), the 2DES maps are remarkably similar in both solvents, showing a distinct checkerboard-like peak pattern^18,28,60–62^ with ~140 meV spacing (Fig. 3a,b, black circles). This pattern is characteristic of coherent vibrational wavepacket motion on the molecule’s excited-state PES, with a dominant period of ~29 fs probably reflecting slight softening of the high-frequency backbone modes in the excited state. At later waiting times, the peak pattern washes out and, in CHX, the map transforms into a broad, featureless SE spectrum along the detection energy. Cross-sections along the excitation energy resemble the linear absorption. Also, in DCM, the 2DES map along the detection energy rapidly transforms into a structureless SE spectrum. After few 100 fs, ESA from $\left|{S}_{1,{\rm{Rel}}}\right\rangle$ appears as an additional negative feature. Interestingly, the early times 2DES maps are hardly affected by solvation. Moreover, their dynamics are clearly different from those expected for wavepacket motion on a displaced harmonic oscillator potential^18,60,61,63^. Typically, in a displaced harmonic oscillator, relaxation damps peak amplitude oscillations with increasing waiting time without affecting the peak pattern along the excitation energy, assuming vibrational relaxation is slower than electronic dephasing. This contrasts with the observations in both solvents, suggesting that excited-state PES anharmonicity of the quadrupolar molecule greatly influences the initial solvation dynamics.

To disentangle the different microscopic contributions to the initial solvation dynamics, we applied the global analysis to examine the Δ*T*/*T* spectra (Fig. 2) in more detail. Subtraction of the slow GSB, SE and ESA contributions with decay times greater than 300 fs (Fig. 2e–h) provides, for CHX, the residual Δ*T*/*T* map shown in Fig. 3c. Beyond the faint, persistent 23 fs ground-state vibrational wavepacket oscillations, the remaining dominant feature is a short-lived SE around the main absorption at 2.1 eV, which decays on a ~50 fs timescale (Fig. 3c, inset). As there is no sizeable Stokes shift between this SE and the absorption, it evidently originates from the Franck–Condon region of the excited-state potential, $\left|{S}_{1}^{* }\right\rangle$. Moreover, given that there is no substantial red-shift with time, the solvation effects seem negligible on this early timescale. We therefore associate the ~50-fs decay of this band with vibrational relaxation of the coherent wavepacket on the excited-state PES. By contrast, in DCM (Fig. 3d), a pronounced, broad SE band—which red-shifts by ~130 meV over ~150 fs (Fig. 3d, black arrow)—remains. Its decay again reflects vibrational relaxation but now on a gradually changing PES induced by rotational orientation of the solvent molecules. In DCM, vibrational coherence thus persists slightly longer than in CHX and the SE band strongly red-shifts. The fast component with vibronically structured spectrum (Fig. 2f, orange) well reproduces much of the overall dynamics of this band. We thus assign its 80 fs decay to vibrational relaxation on the $\left|{S}_{1}\right\rangle$ PES. This SE band thus stems from a vibrationally hot, coherent wavepacket moving on a gradually varying PES due to polar solvation. Subtracting also this fast component from the Δ*T*/*T* reveals coherent vibrational wavepacket motion preceding solvation (Fig. 3d, inset). These initial dynamics closely resemble those in CHX. As in CHX, the residual Δ*T*/*T* persist for ~50 fs and track SE from the Franck–Condon region $\left|{S}_{1}^{* }\right\rangle$. The similarity of residual maps in polar and non-polar solvent strongly suggests that the solvent has little effect on the PES and wavepacket dynamics during the first 50 fs. We can essentially consider the polar solvent as being frozen on this timescale.

Analysis of amplitude and phase of the Δ*T*/*T* oscillations in Fig. 3c,d shows that persistent, fast oscillations with a period of 20–25 fs arise from totally-symmetric A–D–A backbone modes (*Q*_+_) around 1,400–1,450 cm^−1^. Furthermore, the data reveal short-lived oscillations induced by a high-frequency antisymmetric vibration (*Q*_−_) at 1,450 cm^−1^ rapidly decaying within <100 fs in both solvents. In contrast to the *Q*_+_ modes, the phase of the oscillations triggered by the *Q*_−_ mode is independent of the probe energy^64^. As detailed in Supplementary Section 5, this analysis experimentally identifies the *Q*_+_ and *Q*_−_ modes that have been predicted theoretically for this class of quadrupolar dyes^65^. Theoretical models of polar solvation^1,66^ and earlier 2DES studies^67^ suggest an initial inertial phase of the solvation dynamics before random diffusive motion of the solvent sets in. This inertial phase is governed by coherent vibrational motion of solute and/or solvent. As such, the experimental observation of *Q*_−_ may be taken as a manifestation of this inertial phase of solvation in the quadrupolar dye.

### Wavepacket dynamics simulation

To rationalize these observations, we analyse the dynamics within a phenomenological essential-state model^39^ for the low-energy optical properties of quadrupolar dyes^38,39,65^. Building up on Mulliken’s work on donor–acceptor complexes^68^, it considers a charge-neutral state (A–D–A) electronically coupled to two degenerate zwitterionic states (A^−^–D^+^–A and A–D^+^–A^−^) as the relevant electronic excitations. This coupling, which measures the probability of charge transfer between donor and acceptor, delocalizes the electronic excitations across the molecule. The zwitterionic states are each coupled to one high-frequency vibrational stretching mode localized on one arm of the molecule. Symmetric and antisymmetric combinations of this mode yield delocalized effective modes across the molecule, *Q*_+_ and *Q*_−_, respectively. When electronic and vibronic couplings are comparable, yet much smaller than the splitting between charge-neutral and zwitterionic states, their interplay results in a characteristic symmetry-broken lowest excited-state PES ($|{S}_{1}\rangle$). Similar models have been advanced to describe solvent-induced symmetry breaking in symmetric quadrupolar molecules^45,47,48^.

**Fig. 4:**
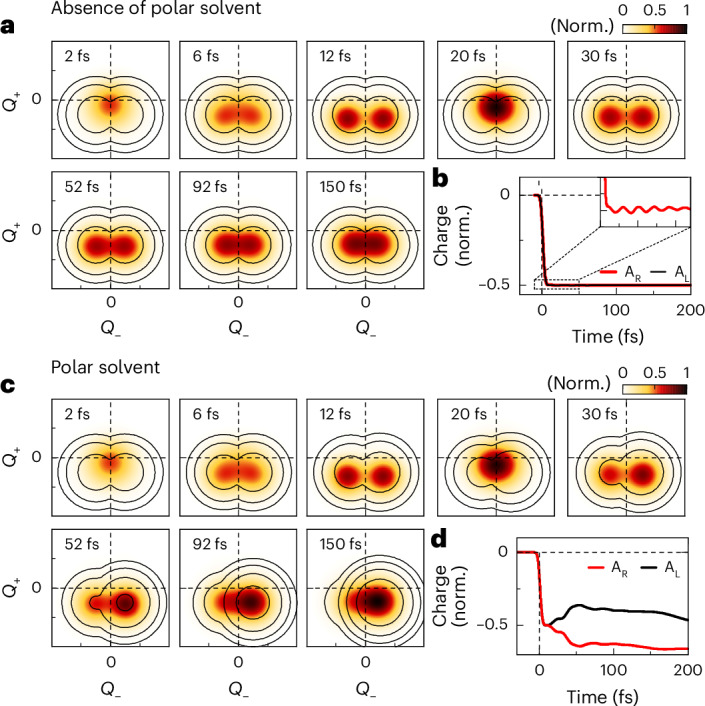
Phenomenological quantum dynamics simulations. **a,c**, Snapshots of the simulated wavepacket dynamics on the excited-state $\left|{S}_{1}\right\rangle$ PES in the dimensionless coordinate plane defined by the *Q*_−_ and *Q*_+_ effective modes in the absence (**a**) and presence (**c**) of a polar solvent. At early times <30 fs, comparable coherent wavepacket motion occurs in both cases, with the wavepacket symmetrically splitting along *Q*_−_. Coherent wavepacket motion persists until vibrational relaxation sets in on a 50 fs timescale. **c**, In the polar solvent, the build-up of the reaction field starts to tilt the PES, lowering the energy of one of the minima towards the solvated state. Black contour lines in **a**,**c** display the double-minimum PES and emphasize the effect of polar solvation (**c**) on the PES. **b,d**, Dynamics of the charge on the right (A_R_) and left (A_L_) acceptor sides of the molecule track charge separation upon optical excitation that forms two antiparallel dipoles in the molecule. **b**, In the absence of polar solvent, the dynamics are modulated only by vibronic coupling to *Q*_+_ which induces faint oscillations of the charge amplitude on the acceptors (inset). **d**, In polar solvent, additionally, the interaction with the building up reaction field results in charge asymmetry between the two arms. This induces a preferential localization of the charge on one arm of the molecule and forms a net dipole across the molecule.

We deduce relevant model parameters from our experiments in CHX, supported by quantum-chemical calculations (Supplementary Section 4). Specifically, we estimate a ~2.1 eV energetic splitting and a 150 meV electronic coupling strength based on the absorption spectrum (Fig. 2a), the Stokes shift and the energy difference between GSB and long-lived SE (Fig. 2c). The frequency of the Δ*T*/*T* coherent oscillations (Fig. 2c), together with quantum-chemical calculations, set a 178 meV energy for the C–C stretching mode. The relative amplitude of vibronic peaks in the emission spectrum (Fig. 2a) provides a Huang–Rhys factor of ~0.3, confirmed by quantum-chemical calculations (Supplementary Figs. 16 and 20). These parameters indeed result in comparable electronic and vibronic coupling strengths, yielding an anharmonic, symmetry-broken $|{S}_{1}\rangle$ PES with a double-minimum along *Q*_−_ and displaced along *Q*_+_ (Fig. 4a, contour lines). This double-minimum PES implies a local polar nature in each minimum^39^, which then enables polar solvation to alter it.

Impulsive resonant photoexcitation of the system launches coherent wavepacket motion on the saddle point $\left|{S}_{1}^{* }\right\rangle$ of the excited-state PES (Figs. 4a and 1a) centred at ${Q}_{+}={Q}_{-}=0$. The photoinduced transition from the ground to the excited state induces instantaneous charge separation A–D–A → A^−0.5^–D^+1^–A^−0.5^, creating two identical antiparallel dipoles in the molecule and a symmetric charge distribution in the excited state (Figs. 4b and 1b). Driven by vibronic couplings, the wavepacket oscillates along *Q*_+_ while symmetrically splitting along *Q*_−_ toward the PES’s minima (Fig. 4a), periodically refocusing when returning to the anharmonic region^65^. This periodic wavepacket splitting and refocusing persists until vibrational relaxation damps the coherent motion while concurrently relaxing the vibronically hot population (Fig. 4a) on a 50 fs timescale. This timescale is deduced from Δ*T*/*T* and 2DES. The motion on the anharmonic PES explains the vibronic resonances observed in Δ*T*/*T* and 2DES in both solvents for <50 fs (Fig. 3), and the observed deviation from a harmonic wavepacket motion. Importantly, this coherent motion reflects excited-state symmetry breaking driven by vibronic couplings of the A–D–A backbone in the absence of solvation^65^. The simulated charge dynamics on each acceptor site (Fig. 4b) reveal faint charge oscillations arising from vibronic coupling to *Q*_+_ (Fig. 4b, inset).

Now we introduce the effect of polar solvation within a reaction field approach^39^, considering a continuum model that approximates the dynamical solvent response as a single exponential^2^ (Supplementary Section 3). The reaction field dynamically modifies the coupling between the zwitterionic states along *Q*_−_ on the solvation timescale, altering the excited-state PES along *Q*_−_. The Stokes shifts in our linear spectra (Fig. 2a,b) provide an estimation for the equilibrium value of the reaction field. Our experimental results and characteristic solvent relaxation times of DCM^59^ indicate a 500 fs rise time of the reaction field. The simulations reveal that the $|{S}_{1}\rangle$ PES is essentially unchanged by the solvent polarity at early times (Fig. 4c), since the reaction field is too weak to considerably alter the molecule’s eigenstates. As such, initial wavepacket dynamics are largely unaffected by the polar solvent (Fig. 4c) yet governed by intramolecular vibronic couplings. This rationalizes the similar 2DES vibronic features and Δ*T*/*T* spectra in both solvents at early times (Fig. 3a,b and insets in Fig. 3c,d). Only beyond the first vibrational period, the build-up of the reaction field induces a gradual tilting of the excited-state PES along *Q*_−_ (Fig. 4c) and wavepacket localization preferentially on one side (Fig. 4c). Concurrently, this leads to dynamical charge localization on one arm of the molecule (Fig. 4d; refer also to Fig. 1b and Supplementary Figs. 13 and 14), forming a significant excited-state dipole. Quantum-chemical simulations (Supplementary Table 1) suggest that the dipole moment increases from about 2 to 15 Debye due to this localization. The PES energy lowering explains the rapid red-shift in the experimental nonlinear spectra in DCM (Fig. 3d, arrow), confirmed by our quantum-chemical modelling (Supplementary Table 2).

We evaluate the electronic coherence between local zwitterionic states to further analyse the timescale of symmetry-breaking charge transfer^48,69,70^ (Supplementary Fig. 22). In the absence of polar solvent, electronic coherence rapidly decays within ~50 fs and is modulated by ~20 fs oscillations reflecting vibronic coupling to *Q*_−_. A finite coherence remains, consistent with wavepacket localization in the symmetry-broken S_1_ PES. In the polar solvent, very similar initial decay and oscillation pattern occur within ~50 fs, indicating that vibronic couplings dominate the symmetry-breaking dynamics on this timescale. Only beyond ~50 fs, the coherence loss is accelerated by solvent-induced symmetry breaking. This indicates that decoherence proceeds on two timescales. It starts before solvation, governed by intramolecular vibronic couplings, and is subsequently enhanced by the onset of polar solvation, in agreement with our experimental observations.

## Discussion

Vibronic couplings to high-frequency *Q*_+_ and *Q*_−_ modes are central components of essential states models^39^. Our analysis of the Δ*T*/*T* oscillations (Supplementary Section 5) confirm such couplings experimentally. This suggests that a symmetry-broken PES—illustrated in Fig. 1—governs the initial quantum dynamics and photoinduced charge separation in this prototypical quadrupolar dye. It also reveals that the assumption of a single *Q*_+_ and *Q*_−_ mode (Fig. 1) is an oversimplification, whereas higher-dimensional PES^69^ resulting from multimode couplings are needed for a more realistic description.

Our study distinguishes two types of symmetry breaking: (1) intramolecular vibronic coupling to *Q*_−_, which creates a double-minimum excited-state PES (|*S*_1_〉) (Fig. 1a), and (2) solvent-induced symmetry breaking, where the interaction of the molecule with the polar solvent localizes the charge on one arm. The first is an intrinsic, theoretically predicted^39^ effect of intramolecular vibronic coupling that does not lead to preferential charge localization and thus cannot be inferred from the charge distribution of |*S*_1_〉. By contrast, the second directly affects the charge distribution across the molecule backbone, forming a dipolar excited state |*S*_1,Sol_〉 with the charge preferentially on one molecular arm (Fig. 1b). Often, only solvent-induced charge localization is considered as excited-state symmetry breaking^50^. Here, our results highlight vibronic coupling to *Q*_−_ resulting in a distinct type of symmetry breaking that manifests as the formation of a double-minimum potential (Figs. 1a and 4a, contour lines).

In conclusion, we have provided evidence that intramolecular vibronic couplings to high-frequency C–C stretching modes dominate the initial sub-50-fs photoinduced charge-transfer dynamics in a prototypical quadrupolar molecule. This results from a double-minimum, symmetry-broken excited-state PES induced by comparable electronic coupling between the two molecular arms and vibronic coupling to C–C vibrations in each arm. Vibronic couplings to a high-frequency antisymmetric mode, characteristic for this class of symmetry-broken PES, are identified by analysing the phase of coherent excited-state wavepackets.

Following optical excitation, two equal but antiparallel dipoles form across the molecule; these initially freeze the solvent response. Subsequently, the onset of polar solvation starts to influence the PES, thus supporting symmetry breaking initiated by vibronic couplings. The interplay of vibronic-coupling-driven symmetry breaking and solvation ultimately leads to preferential charge localization on one molecular arm and, hence, a polar excited state. The latter accounts for the pronounced emission solvatochromism observed in these molecules.

These conclusions are supported by recent transient absorption studies of symmetry-breaking charge transfer in slip-stacked perylenediimide dimers and trimers^52^. In these systems, vibronic coupling to high-frequency carbon backbone modes facilitates coherent mixing between Frenkel excitons and charge-transfer states, which gradually decay into symmetry-broken charge-separated states due to solvent fluctuations and coupling low-frequency molecular vibrations.

Our results provide crucial new insight into the initial stages of photoinduced intramolecular charge-transfer dynamics in quadrupolar molecules, revealing that intramolecular vibronic couplings drive excited-state symmetry breaking preceding the onset of solvation. Applications of these quadrupolar molecules in organic electronics, for example, solar cells^71,72^, or as a material platform to explore nanoscale coherent charge transport, commonly involve their use in solid-state form. In the solid-state, high-frequency bond vibrations, such as C–C stretching motions, are typically not constrained by steric interactions. Furthermore, intermolecular interactions in *π*–*π*-stacked systems are expected to facilitate excitation delocalization^18,20,73^. We therefore anticipate that such high-frequency intramolecular vibrations may play a central role in aggregates of quadrupolar molecules. As such, the design of efficient molecular building blocks for optoelectronic applications requires detailed understanding of their light-induced ultrafast dynamics, of the interplay between intramolecular and intermolecular couplings in solid-state structures, along with the interaction with the dielectric environment^74^. Our results elucidate the complex interplay of these phenomena and thus may have direct implications toward controlling coherent charge transport in nanostructures.

## Methods

### 2,2′-{{[4-(2-Hexyldecyl)-4*H*-dithieno[3,2-*b:*2′,3′-*d*]pyrrol-2,6-diyl]bis[3,4′-dihexyl-(2,2′-bithiophene)-5′,4-diyl]}bis(methaneylylidene)}bis(1*H*-inden-1,3(2*H*)-dione) (3)

**Extended Data Fig. 1:**
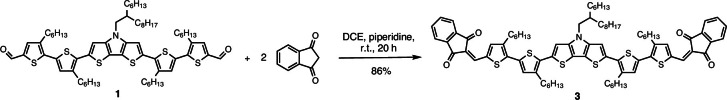
Scheme of the synthesis of the A–D–A molecule. Synthesis of Compound **3**, the investigated A–D–A molecule. Synthesized from bisaldehyde (indicated as compound **1**) and five equivalents of 1,3-indandione.

Compound **3** is synthesized in a Knoevenagel condensation from bisaldehyde (indicated as compound **1** in Extended Data Fig. 1) and five equivalents of 1,3-indandione. Specifically, 159.7 mg (0.14 mmol) of bisaldehyde and 103.8 mg (0.71 mmol) of 1,3-indandione are dissolved in 20 ml of dichloroethane and a few drops of piperidine are added as catalyst. The solution is stirred at room temperature for 20 h. After aqueous workup and final washing with saturated sodium chloride solution, the organic phase is dried over magnesium sulfate. Following column chromatographic purification, the A–D–A molecule (compound **3** in Extended Data Fig. 1) is obtained as a dark blue powder with a yield of 86%.

Safety note: dichloroethane is a suspected carcinogen and should be used in a fume hood. Piperidine is corrosive and should be handled with appropriate protective equipment.

### Compound characterization

Nuclear magnetic resonance spectra were recorded on a Bruker DRX 400 using residual solvents signals as the internal standard. High-resolution mass spectra were recorded using Fourier-transform ion cyclotron resonance mass spectrometry. ^1^H-NMR and high-resolution mass spectra results are reported in Supplementary Section 7 and shown in Supplementary Figs. 3 and 4.

To characterize the electrochemical properties in the full molecular construct, we have recorded the cyclic voltammogram of our molecule in solution to determine the redox potentials and the energy levels of the frontier orbitals. Cyclic voltammetry is performed on the molecule in dichloromethane/tetrabutylammonium hexafluorophosphate (0.1 M) with a scan speed of 100 mV s^−1^ at room temperature and referenced against Fc/Fc^+^. The data are shown in Supplementary Fig. 5. Two reversible oxidation waves at *E*_ox_^1^ = 0.27 V and *E*_ox_^2^ = 0.65 V are observed, which can be assigned to the stepwise oxidation of the conjugated backbone with formation of radical cations and dications arising from the donor core. In the negative potential part of the cyclic voltammogram, one irreversible reduction wave at *E*_red_^1^ = −1.34 V corresponds to the reduction of the acceptor units. These redox potentials result in the following energies of the frontier molecular orbitals *E*_HOMO_ = −5.26 eV and *E*_LUMO_ = –3.88 eV, calculated from the onset of the first oxidation and reduction wave. Their difference *E*_g_^CV^ = |*E*_HOMO_| − |*E*_LUMO_| = 1.38 eV yields the electrochemical gap.

The Raman spectrum (Supplementary Fig. 2) of the A–D–A molecule in powder form is recorded using a WITec alpha 300R Raman Microscope with a UHTS-300 spectrometer. The excitation wavelength is of 532 nm.

### Sample preparation

To prepare the solutions used for the experiments, the A–D–A molecule is dissolved either in CHX or DCM. The concentration is kept to <1 mg ml^−1^ in both solvents to prevent any possible aggregation. The linear absorption spectra of the A–D–A molecule dissolved in CHX and DCM are recorded with a Shimadzu SolidSpec 3700 spectrometer. Photoluminescence spectra are recorded in a home-built set-up by exciting the samples with the 532 nm (~2.33 eV) line of a narrowband laser diode. The excitation beam is focused into the sample cuvette with a microscope objective (numerical aperture NA = 0.4, 20×). A second microscope objective (NA = 0.4, 25×) collects the emitted light under an angle of 90° with respect to the incident beam and focuses it into a fibre spectrometer (OceanOptics USB2000+). For the photoluminescence measurements, the solutions are prepared with an absorbance of <0.1 in a 10 mm path length to minimize reabsorption by the sample.

### Ultrafast pump–probe and two-dimensional electronic spectroscopy set-up

We use a home-built partially collinear 2DES set-up as reported previously^28,32^. Broadband optical pulses, with a spectrum ranging from roughly 1.7 eV to 2.4 eV (Supplementary Fig. 1a), are generated in a home-built non-collinear optical parametric amplifier (NOPA) pumped by the second-harmonic of a regeneratively amplified Ti:sapphire laser (Spectra Physics Spitfire Pro, central wavelength 800 nm, ~140 fs pulse duration, repetition rate 5 kHz). Chirped mirrors (Laser Quantum DCM9) placed after the NOPA are used to compress the pulses. Second-harmonic generation frequency resolved optical gating of the cross-correlation between pump and probe arms, recorded at the sample position, indicates a time-resolution of ~8 fs (Supplementary Fig. 1b). This includes a 1 mm quartz window to account for and pre-compensate the dispersion introduced by the cuvette containing the sample. A broadband beam splitter divides the beam into pump and probe arms after the NOPA. For the 2DES measurements, a pair of time-delayed, phase-locked collinear pump pulses are generated by a common path interferometer based on birefringent wedges (TWINS)^75^. The time delay between the pump pulses—that is, the coherence time *τ*—is controlled by a motorized translation stage (Physik Instrumente M122.2DD). The dispersion introduced by the wedges in the TWINS is compensated by means of an additional pair of chirped mirrors (Laser Quantum DCM10) placed in the pump arm. A small fraction of the pump beam is sent to a photodiode to record the autocorrelation of the two pump pulses on-the-fly during the measurements, which is used to calibrate the coherence time axis^75^. The time delay between the second pump pulse and probe pulse—that is, the waiting time *T*—is controlled by a motorized translation stage (Physik Instrumente M111.1DG) in the probe arm. A spherical mirror is used to focus pump and probe beams to a spot size of 90 × 70 µm^2^ into a quartz cuvette (Hellma Macro-cuvette 110-QS, 1 mm path length) containing the A–D–A molecules in solution. The spot size of the pump and probe beams at the sample position are characterized by a beam camera (Thorlabs DCC1545M-GL). All measurements are performed with pump and probe fluences of 70 µJ cm^−^^2^ and 55 µJ cm^−^^2^, respectively. The relative polarization between the linearly polarized pump and probe pulses is set to 55°. After the sample, the transmitted probe beam is dispersed into a monochromator (Princeton Instruments, Acton SP2150i) and recorded with a high-speed CCD line camera (e2v AviiVa EM4), whereas the transmitted pump beam is blocked. Differential transmission spectra $\frac{\Delta T\left(\tau, T,{E}_{\rm{D}}\right)}{T\left(\tau ,T,{E}_{\rm{D}}\right)}=\frac{{I}_{\rm{on}}\left(\tau ,T,{E}_{\rm{D}}\right)-{I}_{\rm{off}}\left(\tau ,T,{E}_{\rm{D}}\right)}{{I}_{\rm{off}}\left(\tau ,T,{E}_{\rm{D}}\right)}$ are recorded as a function of the time delays *τ* and *T*, and the detection energy *E*_D_. The transmitted probe spectra though the sample with the pump beam being switched on and off by a mechanical chopper at a frequency of 2.5 kHz are denoted as *I*_on_ and *I*_off_, respectively. To obtain absorptive 2DES maps, *A*_2D_(*E*_X_,*T*,*E*_D_), we take the real part of the Fourier-transform of the measured Δ*T*(*τ*, *T*, *E*_D_)*/*$T$(*τ*, *T*,*E*_D_) signal along the coherence time obtaining the excitation energy *E*_X_. The two-pulse differential transmission measurements are performed by setting *τ* = 0 fs. Both the absorbance of the samples and the pump-fluence in the pump–probe and 2DES experiments are kept as low as possible to ensure meaningful signals while maintaining a good signal-to-noise ratio.

### Subtraction of the solvent response

The off-resonant excitation of the solvent with a short laser pulse gives rise to a purely electronic cross-phase modulation (XPM)^76^ nonlinearity during the pump–probe overlap. It arises from the time-dependent nonlinear refractive index of the solvent that follows the local pump intensity and leads to transient oscillations of the Δ*T*/*T* spectra. In addition, the pump off-resonantly drives coherent vibrational wavepacket motion in the electronic ground state through impulsive stimulated Raman scattering (ISRS)^77^. This gives rise to persistent oscillations at the frequencies of the Raman modes of the solvent. Two methods are used to subtract these undesired solvent contributions from the pump–probe data. First, we subtract the Δ*T*/*T* dynamics of a reference measurement of the neat solvent, performed under the same experimental conditions as those of the A–D–A molecules. As this methodology has been viewed skeptically^76^, we also perform simulations of the XPM and ISRS signal. For XPM, we use an analytical model developed in ref. ^78^ and validated in ref. ^76^. For the ISRS contributions, we assume damped phase-shifted cosine functions for the response of each Raman mode of the solvent. These response functions are convoluted with the measured instrument response function of the set-up. More details are discussed in Supplementary Section 1. Within our measurement accuracy, both methods give identical results. All Δ*T*/*T* spectra shown in the manuscript are obtained after subtracting the reference measurements of the neat solvent. Also all 2DES data have been corrected for the neat solvent response analogously to the Δ*T*/*T* data. Subtraction of the solvent response from the 2DES of the quadrupolar molecule for an exemplary early waiting time of 10 fs is shown in Supplementary Fig. 8 demonstrating that the 2DES peak pattern is not significantly influenced by the solvent-induced XPM even at early times.

### Phenomenological quantum dynamics simulations

To simulate the quantum dynamics of an A–D–A molecule in solution, we use the essential-state model introduced by Painelli and co-workers^39,65^. This model considers the A–D–A molecule as being composed of two donor–acceptor dipoles, resulting in a charge-neutral ground state $|N\rangle =|{\rm{ADA}}\rangle$ and two zwitterionic states $\left|{Z}_{1}\right\rangle =\left|{{\rm{A}}}^{-}{\rm{D}}^{+}{\rm{A}}\right\rangle$ and $\left|{Z}_{2}\right\rangle =\left|A{\rm{D}}^{+}{\rm{A}}^{-}\right\rangle$. The zwitterionic states are separated from the $|N\rangle$ state by an energy gap $\eta$ and they are electronically coupled to it with a coupling strength $t$. Charge redistribution from $|N\rangle$ to the $\left|{Z}_{1}\right\rangle$ or $\left|{Z}_{2}\right\rangle$ state in each arm of the molecule is accounted for by introducing two independent effective dimensionless coordinates^39^ *Q*_1_ and *Q*_2_, respectively, which are associated with a high-frequency vibrational mode. Vibronic coupling results in displacements *λ*_*dia*_ in the PES of the excited states along these dimensionless coordinates^40^. We take the C–C stretching mode, with an energy of $\hslash {\omega }_{\rm{vib}}=178$ meV, as the dominant vibration for each coordinate according to our experiments and quantum-chemical simulations. Following ref. ^39^, the zwitterionic states can be expressed as symmetric and antisymmetric combinations of $\left|{Z}_{+}\right\rangle =\frac{\left(\left|{Z}_{1}\right\rangle +\left|{Z}_{2}\right\rangle \right)}{\sqrt{2}}$ and $\left|{Z}_{-}\right\rangle =\frac{\left(\left|{Z}_{1}\right\rangle -\left|{Z}_{2}\right\rangle \right)}{\sqrt{2}}$. Analogously, symmetric ${Q}_{+}=\frac{\left({Q}_{1}+{Q}_{2}\right)}{\sqrt{2}}$ and antisymmetric ${Q}_{-}=\frac{\left({Q}_{1}-{Q}_{2}\right)}{\sqrt{2}}$ coordinates are defined^39^. Details on the model Hamiltonian are reported in Supplementary Section 3. To simulate the quantum dynamics, we numerically solve the master equation for the density matrix in the Lindblad form^79^ using a nonperturbative approach^80^

1 $$\frac{d\rho }{{dt}}=-\frac{i}{\hslash }\left[\left({H}_{0}+{H}_{i}\right),\rho \right]+{\mathscr{L}}\left(\;\rho \right)$$

where $${H}_{0}={H}_{\rm{mol}}+{H}_{\rm{sol}}$$ is the total system Hamiltonian describing the free evolution of the system and the effect of the solvent as defined in Supplementary equations (5) and (7) in Supplementary Section 3, respectively. The light–matter interaction of the system is modelled in semi-classical point-dipole approximation as $${H}_{i}(t)=$$$$-{\mu }_{0}E(t)(|{Z}_{+}\rangle \langle {Z}_{-}|+|{Z}_{-}\rangle \langle {Z}_{+}|)$$. It is important to note that $${|N}\rangle$$, $$\left|{Z}_{+}\right\rangle$$ and $$\left|{Z}_{-}\right\rangle$$ are not eigenstates of the system. The eigenstates, which we denote $$|{S}_{0}\rangle$$, $$\left|{S}_{1}\right\rangle$$ and $$\left|{S}_{2}\right\rangle ,$$ are superpositions of $${|N}\rangle$$, $$\left|{Z}_{+}\right\rangle$$ and $$\left|{Z}_{-}\right\rangle$$ according to Supplementary equation (5), and can be obtained by numerical diagonalization of the Hamiltonian in Supplementary equation (5). Accordingly, the light–matter interaction in the basis of eigenstates, describing optical transitions between $$|{S}_{0}\rangle$$, $$\left|{S}_{1}\right\rangle$$ and $$\left|{S}_{2}\right\rangle$$ can be obtained by applying a basis transformation to the one defined above for the $${|N}\rangle$$, $$\left|{Z}_{+}\right\rangle$$, $$\left|{Z}_{-}\right\rangle$$ basis. To model the dynamics, in the simulations we set the initial state as $$|{S}_{0}\rangle$$, the ground state of the system. The external optical field exciting the system is taken as a Gaussian pulse $$E\left(t\right)=$$$$A{e}^{-2\mathrm{ln}2{\left(\frac{t}{{t}_{w}}\right)}^{2}}\cos \left({\omega }_{0}t\right)$$ with carrier energy $$\hslash {\omega }_{0}=2.2{\rm{eV}}$$ and pulse duration *t*_*w*_ = 5 fs. We introduce vibrational relaxation phenomenologically with the Lindblad superoperator $${\mathscr{L}}\left(\,\rho \right)=$$$${\sum }_{m=+,-}{\gamma }_{m}\left(2{L}_{m}\rho {L}_{m}^{\dagger }-{L}_{m}^{\dagger }{L}_{m}\rho -\rho {L}_{m}^{\dagger }{L}_{m}\right)$$, where *γ*_+_ (*γ*_−_) describes the relaxation rate along *Q*_+_ (*Q*_−_), and the operators *L*_+_ = *b*_+_ and *L*_−_ = b_−_ are the annihilation operators of the harmonic oscillator for mode *Q*_+_ and *Q*_−_, respectively. Effects of a finite solvent temperature are neglected. We set *γ*_+_ = *γ*_−_ for simplicity. In the basis of vibronic states $$\left|i,k\right\rangle$$, with $$i=\mathrm{1,2,3}$$ denoting the electronic states $$|N\rangle$$, $$\left|{Z}_{+}\right\rangle$$ and $$\left|{Z}_{-}\right\rangle$$, respectively, and $$k=\mathrm{0,1},\ldots,{N}_{V}-1$$, the vibrational quantum number of the two-dimensional harmonic oscillator wavefunction with *N*_V_ the total number of vibrational quanta considered, the density matrix is defined as $$\rho ={\sum }_{i=1}^{3}{\sum }_{j=1}^{3}{\sum }_{k=0}^{{N}_{V}-1}{\sum }_{l=0}^{{N}_{V}-1}{c}_{i,k}{c}_{j,l}^{* }\left|i,k\right\rangle \left\langle j,l\right|$$. The wavepacket dynamics following photoexcitation is calculated by computing the probability density in the (*Q*_+_, *Q*_−_) coordinate plane^65^ as

2 $$P\left({Q}_{+},{Q}_{-},t\right)=\mathop{\sum }\limits_{i=1}^{3}\mathop{\sum }\limits_{k=0}^{{N}_{V}-1}\mathop{\sum }\limits_{l=0}^{{N}_{V}-1}{\rho }_{i,k,i,l}\left(t\right){\varPsi }_{k}\left({Q}_{+},{Q}_{-}\right){\varPsi }_{l}^{* }\left({Q}_{+},{Q}_{-}\right)$$

where ${\rho }_{i,k,i,l}=\left\langle i,k\left|\rho \right|i,l\right\rangle ={c}_{i,k}^{* }{c}_{i,l}$ and ${\varPsi }_{k}\left({Q}_{+},{Q}_{-}\right)$ are two-dimensional harmonic oscillator wavefunctions along the two independent vibrational coordinates *Q*_+_ and *Q*_−_. The expectation values $\left\langle \hat{\sigma }\right\rangle ={\sum }_{i=2}^{3}{\sum }_{k=0}^{{N}_{V}-1}{\left|{c}_{i,k}\right|}^{2}$ and $$\left\langle \hat{\delta }\right\rangle ={\sum }_{i=2}^{3}{\sum }_{\begin{array}{c}\mathrm{j}=2\\ \mathrm{j}\ne i\end{array}}^{3}{\sum }_{k=0}^{{N}_{V}-1}{c}_{i,k}{c}_{j,k}^{* }$$ give the charge dynamics on the donor and the charge difference between the two acceptor moieties (see Supplementary equation (6a,b) for the definition of the operators). The charge on the donor is thus given by the sum of the electronic population of $\left|{Z}_{+}\right\rangle$ and $\left|{Z}_{-}\right\rangle$, whereas electronic coherence between these states governs the charge unbalance on the two acceptors. The total charge on both acceptors is ${C}_{\rm{A}_{\rm{L}}}\left(t\right)+{C}_{\rm{A}_{R}}\left(t\right)=-{C}_{\rm{D}}\left(t\right)=-\left\langle \hat{\sigma }\right\rangle$ and their difference is ${C}_{\rm{A}_{L}}\left(t\right)-{C}_{\rm{A}_{R}}\left(t\right)=\left\langle \hat{\delta }\right\rangle$, where ${C}_{\rm{A}_{L}}$ and ${C}_{\rm{A}_{R}}$ are the charges on the left and on the right acceptor, respectively, *C*_D_ is the charge on the donor, and $\left\langle \cdot \right\rangle ={Tr}\left\{\cdot \rho \right\}$ denotes the expectation value of the operator. We obtain

3a $${C}_{\rm{A}_{L}}(t)=\frac{\langle \hat{\sigma }\rangle +\langle \hat{\delta }\rangle }{2}=\frac{1}{2}\mathop{\sum }\limits_{i=2}^{3}\mathop{\sum }\limits_{k=0}^{{N}_{V}-1}{|{c}_{i,k}|}^{2}+\frac{1}{2}\mathop{\sum }\limits_{i=2}^{3}\mathop{\sum }\limits_{\begin{array}{c}j=2 \\ j\ne i\end{array}}^{3}\mathop{\sum }\limits_{k=0}^{{N}_{V}-1}{c}_{i,k}{c}_{j,k}^{\ast }$$

3b $${C}_{\rm{A}_{R}}(t)=\frac{\langle \hat{\sigma }\rangle -\langle \hat{\delta }\rangle }{2}=\frac{1}{2}\mathop{\sum }\limits_{i=2}^{3}\mathop{\sum }\limits_{k=0}^{{N}_{V}-1}{|{c}_{i,k}|}^{2}-\frac{1}{2}\mathop{\sum }\limits_{i=2}^{3}\mathop{\sum }\limits_{\begin{array}{c}j=2\\ j\ne i\end{array}}^{3}\mathop{\sum }\limits_{k=0}^{{N}_{\rm{V}}-1}{c}_{i,k}{c}_{j,k}^{\ast }$$

The expressions in equation (3a,b) emphasize that the formation of a net dipole in the molecule is accompanied by a change in electronic coherence between $\left|{Z}_{+}\right\rangle$ and $\left|{Z}_{-}\right\rangle$.

### Quantum-chemical calculations

To theoretically evaluate geometries, electronic structures and vibronic properties of the molecule in different solvents, we perform quantum-chemical calculations using the CAM-B3LYP^81^ functional and the def2-SVP^82^ basis set as implemented in the Gaussian 16 package^83^. Solvent effects are incorporated via the polarizable continuum model within the self-consistent reaction field scheme. The polarizable continuum model equilibrium solvation^84^ is used in the ground-state geometry optimizations. Time-dependent density function theory (TD-DFT) calculations are further performed to compute electronically excited-state properties. The TD-DFT method combined with equilibrium linear-response solvation^85^ is further adopted to optimize the first excited-state geometry. State-specific solvation methods, that is, the corrected linear-response^86^ and the external iteration^87^ technique, are then employed to compute the first excited-state energies of the relaxed ground- and excited-state geometries. Obtained transition density matrices form TD-DFT simulations associated with electronically excited states were further decomposed into distributions of the electron and hole densities as implemented in the Multiwfn package^88^. The calculated S_1_ and S_2_ states qualitatively agree with the electronic excitations suggested by the essential-state model in terms of their optical activity and charge redistribution shown by the natural transition orbital plots in Supplementary Fig. 14. Real space orbital distributions of these electron and hole densities are then plotted in Fig. 1b as well as Supplementary Fig. 13. We note that the NTOs shown in Supplementary Fig. 14 better reflect the differences in geometry and electronic distribution between the S_1_ and S_1_* states.

## Online content

Any methods, additional references, Nature Portfolio reporting summaries, source data, extended data, supplementary information, acknowledgements, peer review information; details of author contributions and competing interests; and statements of data and code availability are available at 10.1038/s41557-025-01908-7.

## Supplementary information

Supplementary Information Supplementary Text, Tables 1-4, Figs. 1–32 and References.

Supplementary Data 1 Optimized ground-state geometry in cyclohexane.

Supplementary Data 2 Optimized ground-state geometry in dichloromethane.

Supplementary Data 3 Optimized S1 excited-state geometry in cyclohexane.

Supplementary Data 4 Optimized S1 excited-state geometry in dichloromethane.

## Data Availability

The data that support the findings of this study are presented in the manuscript and Supplementary Information in graphical form. Datasets underlying the results presented in the manuscript are available at 10.5281/zenodo.15780553 (ref. ^89^), and from the authors on reasonable request.

## Code availability

Numerical codes used in this study are based on MATLAB routines and Gaussian 16, as described in detail in the Methods and Supplementary Information. Parts relevant to reproducing the results of this work can be made available from the authors on reasonable request.
