# Supplementary material for: Vibronic coupling-driven symmetry breaking and solvation in the photoexcited dynamics of quadrupolar dyes
Source: Nat Chem. 2025 Aug 20;17(11):1742–9. doi: 10.1038/s41557-025-01908-7 (PMC12580337; doi:10.1038/s41557-025-01908-7)
Supplement: Supplementary file 1 — Supplementary Text, Tables 1-4, Figs. 1–32 and References. [file 41557_2025_1908_MOESM1_ESM.pdf]

nature chemistry

Supplementary information https://doi.org/10.1038/s41557-025-01908-7

Vibronic coupling-driven symmetry breaking and solvation in the photoexcited dynamics of quadrupolar dyes

In the format provided by the authors and unedited

Table of Contents

Supplementary Text 2

1. Solvent subtraction from the differential transmission data 2

2. Global analysis of the differential transmission data 4

3. Definition of the model Hamiltonian and phenomenological potential energy surfaces ... 6

4. Quantum chemical calculations 8

4.1 Molecular geometry and permanent dipole moment. 8

4.2 Excited state properties. 9

4.3 Orbital representation of transition densities and electron-hole distributions as well as natural transition orbitals of the S1 state calculated for the GS and S1 geometries. 9

4.4 Huang-Rhys factors. 9

4.5 Vibronic absorption and emission spectra. 10

5. Analysis of the temporal oscillations in the experimental $\Delta T/T$ data 10

5.1 Signatures of symmetric and antisymmetric modes in CHX. 11

5.2 Signatures of symmetric and antisymmetric modes in DCM. 12

5.3 Polarization-resolved pump-probe measurements. 14

6. Time-resolved fluorescence of the A-D-A molecule in CHX and DCM 16

7. Compound characterization data 17

Supplementary Tables 18

Supplementary Figures 20

References 37

Supplementary Text

1. Solvent subtraction from the differential transmission data

The off-resonant excitation of a solvent with a short laser pulse gives rise to a purely electronic cross-phase modulation (XPM)[1,2] nonlinearity during the pump-probe overlap. XPM arises from the time-dependent nonlinear refractive index of the solvent that follows the local pump intensity and leads to transient spectral oscillations in the $\Delta T/T$ spectra. In addition, the pump off-resonantly drives coherent vibrational wavepacket motion in the electronic ground state through impulsive stimulated Raman scattering (ISRS)[3]. This induces persistent temporal oscillations at the frequencies of the dominant Raman modes of the solvent. Here we use two methods to subtract these undesired solvent contributions from the $\Delta T/T$ data.

First, we subtract the $\Delta T/T$ dynamics of a reference measurement of the neat solvent, performed under the same experimental conditions as the measurements on the A-D-A molecules. $\Delta T/T$ spectra of the neat CHX and DCM solvents are shown in Supplementary Figs. 6b and 7b, respectively. In both solvents, the $\Delta T/T$ maps exhibit (i) XPM at waiting times of less than 100 fs, and (ii) persistent amplitude oscillations arising from ISRS over the entire investigated timescale up to 1 ps[4,5]. To extract the frequencies of the dominant Raman modes of the solvent, we show the amplitudes of the Fourier transforms (FT) of the $\Delta T/T$ transients at selected probe energies for CHX in Supplementary Fig. 6g and for DCM in Supplementary Fig. 7g (black lines). In CHX, the FT reveals a prominent Raman mode at 810 cm^{-1}, whereas in DCM, three Raman modes are identified at 290 cm^{-1}, 710 cm^{-1} and 790 cm^{-1}. The deduced mode frequencies are in good agreement with known Raman modes of these solvents[6,7].

Since solvent removal by subtraction of reference measurements has been discussed critically in the literature[1], we compare this direct subtraction method to a second, alternative procedure[1] that is based on the subtraction of simulated ISRS and XPM signals and their contributions to the $\Delta T/T$ spectra. For the ISRS contribution, we describe the response of each of the solvent modes by a damped, phase-shifted cosine function, $R(t) = \Theta(t)\ \sum_{j=1}^{n} A_j\, cos(\omega_j t + \phi_j) e^{-\gamma_j t}$. Here, A_j, ω_j, ϕ_j and γ_j are the amplitude, frequency, phase, and damping rate of the j-th vibrational mode of the solvent, respectively. In agreement with the measured $\Delta T/T$ of the neat solvents (Supplementary Figs. 6g and 7g), we consider $n = 1$ Raman modes for CHX and $n = 3$ for DCM. At each energy E_D in the $\Delta T/T$ map, the ISRS signal is described by the convolution of $R(t)$ with the instrument response function of the setup, which we take as a Gaussian function $I(t) = I_0 \exp(-2\ln(2)\, t^2 / \tau_L^2)$ with a full width at half maximum

(FWHM) of $\sqrt{2}\tau_L$. Here, $\tau_L = 8$ fs denotes the FWHM of the intensity envelopes of the pump and probe pulses, respectively. The pulse durations are deduced from the SHG-FROG cross-correlation measurement shown in Supplementary Fig. 1.

This gives the ISRS signal as a function of waiting time T

$$\mathrm{ISRS}(E_D, T) = \int_{-\infty}^{\infty} R(E_D, t')I(T - t')dt'. \tag{S1}$$

To model the XPM contribution to the $\Delta T/T$, we use an analytical model that has been developed in Ref. 2 and validated in Ref. 1.

$$XPM(E_D, T) = \cos(B(E_D)(T - t_0(E_D))^2 + \phi(E_D)) \cdot \ldots \tag{S2}$$

$$\ldots \left\{ A_0(E_D) \exp\left[-4\ln 2 \left(\frac{T - t_0(E_D)}{\tau_L(E_D)}\right)^2\right] - A_1(E_D)\frac{8\ln 2}{\tau\_L(E_D)^2}(T - t_0(E_D)) \exp\left[-4\ln 2\left(\frac{T - t_0(E_D)}{\tau_L(E_D)}\right)^2\right]\right\}$$

This models the XPM as a sum of a Gaussian with amplitude $A_0(E_D)$ and its first temporal derivative with amplitude $A_1(E_D)$. The parameter $t_0(E_D)$ denotes the time-zero of the XPM trace. Its dependence on the probe energy provides a measure of the chirp introduced on the probe pulses. The parameter $B(E_D)$ and the phase $\phi(E_D)$ are essential for reproducing the XPM fringes. The simulated $\Delta T/T$ map that is obtained by adding the ISRS and XPM contributions, $\Delta T/T = ISRS + XPM$ is shown for CHX in Supplementary Fig. 6c and for DCM in Supplementary Fig. 7c. It demonstrates good agreement with the experimental $\Delta T/T$ map of the neat solvents. We therefore subtract the sum of the simulated ISRS and XPM signals from the experimental $\Delta T/T$ maps of the A-D-A molecule in CHX (Supplementary Fig. 6e) and in DCM (Supplementary Fig. 7e). Very similar $\Delta T/T$ maps are obtained when directly subtracting the independently measured $\Delta T/T$ maps of the neat solvents (Supplementary Figs. 6d and 7d, for CHX and DCM, respectively). To further demonstrate the similarity of these two different methods for subtracting the solvent-related nonlinearities, we show FT traces of the $\Delta T/T$ maps

of the A-D-A molecule in CHX (Supplementary Fig. 6f) and DCM (Supplementary Fig. 7f) at selected probe energies and compare them to the FTs obtained from the measurements of the neat solvents (Supplementary Figs. 6g and 7g). Both methods for the solvent subtraction give nearly identical FT spectra (Supplementary Figs. 6h and 7h). In the main manuscript, we report the $\Delta T/T$ maps of the A-D-A molecules after subtraction of the solvent contribution as obtained from an independent reference measurement of the neat solvents. The resulting maps are shown in Supplementary Figs. 6d and 7d for CHX and DCM, respectively.

2. Global analysis of the differential transmission data

The optical excitation of the A-D-A quadrupolar molecule induces a sequence of intra- and intermolecular relaxation dynamics. To gain insight into these dynamics, we decompose the $\Delta T/T$ time traces at each probe energy E_D into a series of N monoexponential decay components with decay times τ_i

$$\frac{\Delta T}{T}(E_D, t) = \sum_{i=1}^{N} \mathrm{DADS}_i(E_D) \exp\left(-\frac{t}{\tau_i}\right). \tag{S3}$$

The spectral amplitude of each decay component, $DADS_i(E_D)$, is termed Decay Associated Difference Spectrum (DADS)[8]. A global analysis of all $\Delta T/T$ transients was performed using a MATLAB-based toolbox[9].

For the $\Delta T/T$ experiments performed on the A-D-A molecule dissolved in the nonpolar solvent CHX, three major decay components are found with time constants of (i) 40 fs, (ii) 850 fs and (iii) > 10 ps, respectively. We assign these time constants mainly to the following physical processes: (i) intramolecular vibrational relaxation, (ii) energy transfer from the solute to the solvent and (iii) ground state recovery. The DADS spectra that are associated with these components are shown in Supplementary Fig. 10a. For the measurement of the molecule in the polar solvent DCM, the global analysis results in four DADS components with decay times of (i) 20 fs, (ii) 70 fs, (iii) 470 fs, and (iv) > 10 ps. As discussed in the main manuscript, we identify these time constants with (i) the onset of solvation in the polar solvent, i.e., the timescale before polar solvation affects the symmetry-broken excited state potential, (ii) intramolecular vibrational relaxation, (iii) polar solvation and (iv) ground state recovery. The corresponding DADS spectra are depicted in Supplementary Fig. 10b. In both cases, the last time constants are significantly longer than the measurement time window. They thus reflect a component that is too slow to be resolved in our experiments. Oscillatory contributions to the $\Delta T/T$ traces,

stemming from, e.g., coherent vibrational wavepacket dynamics, are not reproduced using such a DADS analysis which focuses only on incoherent relaxation dynamics.

To interpret these decay components and their spectra, commonly evolution-associated difference spectra (EADS) are typically obtained from the DADS as $EADS_k(E_D) = \sum_{i=k}^{N} DADS_i\,(E_D)$ and they are used to extract spectral contributions to the $\Delta T/T$ spectrum. To extract species spectra from EADS, a fundamental assumption is that a sequential rate equation model with substantially different time constants for decay and rise components applies[8]. This assumption, however, does not hold true for our experimental data. Therefore, we use a modified rate equation model to assign the species spectra starting from the DADS as outlined in the following. We assume that photoexcitation triggers a sequential relaxation process in which the population n_i in state i decays exponentially into state $i+1$ at rate $k_i = 1/\tau_i$: $dn_i/\,dt = +\,k_{i-1}n_{i-1} - k_i n_i$, $i = 1, \dots, N$. The resulting populations n_i for $N = 4$ are given as

$$n_1(t) = n_1(0)\exp(-k_1 t), \tag{S4a}$$

$$n_2(t) = \frac{k_1 n_1(0)}{k_1 - k_2}[\exp(-k_2 t) - \exp(-k_1 t)], \tag{S4b}$$

$$\begin{aligned} n_3(t) = \frac{k_1 k_2 n_1(0)}{k_1 - k_2}\Big\{&\left(\frac{1}{k_2 - k_3}\right)[\exp(-k_3 t) - \exp(-k_2 t)] \\ &- \left(\frac{1}{k_1 - k_3}\right)[\exp(-k_3 t) - \exp(-k_1 t)]\Big\}, \end{aligned} \tag{S4c}$$

$$\begin{aligned} &n_4(t) \\ &= \left(\frac{k_1 k_2 k_3 n_1(0)}{k_1 - k_2}\right)\left\{\begin{aligned} &\left(\frac{1}{k_2 - k_3} - \frac{1}{k_1 - k_3}\right)\left(\frac{1}{k_3 - k_4}\right)[\exp(-k_4 t) - \exp(-k_3 t)] + \\ &-\left(\frac{1}{k_2 - k_3}\right)\left(\frac{1}{k_2 - k_4}\right)[\exp(-k_4 t) - \exp(-k_2 t)] + \\ &+\left(\frac{1}{k_1 - k_3}\right)\left(\frac{1}{k_1 - k_4}\right)[\exp(-k_4 t) - \exp(-k_1 t)] \end{aligned}\right\} \end{aligned} \tag{S4d}$$

The population dynamics deduced from the $\Delta T/T$ measurements in CHX and DCM are shown in Fig. 2g,h of the main manuscript, respectively. We use an iterative procedure to deduce the differential transmission spectra $S_i(E_D)$ associated with each "species" i. In general, the final DADS spectrum is $DADS_N = \mathrm{EADS}_N(E_D)$. The N-th decay component in our system has a

time constant $\tau_N = \infty$. In DCM, $DADS_N$ contains GSB from S_0 and ESA from $S_{1,Rel}$, where the spectrum of GSB is known. We thus decompose $\mathrm{DADS}_N(E_D) = GSB(E_D) + ESA(E_D) = S_0 + S_N = \Delta n_0 \overline{S}_0 + n_N(\infty)\overline{S}_N$. For normalization, we set $\Delta n_0 = n_N(\infty)$=1. Subsequently, we deduce the population dynamics $n_N(t)$ of species N from Eq. S4. With this, we can now subtract the contribution of GSB and ESA from the total differential transmission $(\Delta T/T)_{N-1}(E_D, t) = \Delta T/T(E_D, t) - \overline{S}_0 - n_N(t)\overline{S}_N$. This gives a new map, $(\Delta T/T)_{N-1}(E_D, t)$, that contains $N-1$ decay components and allows for retrieving $S_{N-1}(E_D)$. To this aim, the DADS analysis is repeated for the new map resulting in $(\Delta T/T)_{N-1}(E_D, t) = \sum_{i=1}^{N-1} \mathrm{DADS'}_i(E_D) \exp\left(-\frac{t}{\tau_i}\right)$. Now, we extract the last of these new DADS, $\mathrm{DADS'}_{N-1}(E_D) = n_{N-1,0}\overline{S}_{N-1}$. Here the amplitude $n_{N-1,0}$ of the normalized species spectrum $\overline{S}_{N-1}$ is time-independent and follows from the rate equation model. With this we obtain the normalized spectrum of the species $N-1$ as $\overline{S}_{N-1}(E_D) = \mathrm{DADS'}_{N-1}(E_D)/n_{N-1,0}$. We can now calculate the time-dependent population $n_{N-1}(t)$ from (S4). With this we have the time-dependent contribution $n_{N-1}(t)\overline{S}_{N-1}$ to the total differential transmission spectrum. The procedure is repeated until all $S_i(E_D)$ are obtained. The contribution of all incoherent decay components to the differential transmission spectra $(\Delta T/T)_{inc}(E_D, t) = \sum_{i=1}^{N} n_i(t) S_i(E_D)$. The species spectra that have been deduced from the $\Delta T/T$ measurements of the A-D-A molecules are depicted in Figs. 2e,f of the manuscript for the solvent CHX and DCM, respectively.

3. Definition of the model Hamiltonian and phenomenological potential energy surfaces

To construct the relevant potential energy surfaces (PESs) of the A-D-A molecule, we use an essential state model introduced by Painelli and coworkers[10,11]. This model considers the A-D-A molecule as being composed of two donor-acceptor dipoles, resulting in a charge-neutral ground state $|N\rangle = |ADA\rangle$ and two zwitterionic states $|Z_1\rangle = |A^-D^+A\rangle$ and $|Z_2\rangle = |AD^+A^-\rangle$. The zwitterionic states are separated from the neutral state by an energy gap η and they are electronically coupled to it with a coupling strength t. Charge redistribution from $|N\rangle$ to the $|Z_1\rangle$ or $|Z_2\rangle$ state in each arm of the molecule is accounted for by introducing two independent effective dimensionless coordinates[10] Q_1 and Q_2, respectively, which are associated with a high-frequency vibrational mode. Vibronic coupling results in displacements λ_{dia} in the PES of the excited states along these dimensionless coordinates[12]. We take the C-C stretching mode, with an energy of $\hbar\omega_{vib} = 178$ meV, as the dominant vibration for each coordinate according to our experiments and quantum chemical simulations. Following Ref. [10], the zwitterionic states can be expressed as symmetric and antisymmetric combinations of $|Z_+\rangle = \frac{(|Z_1\rangle + |Z_2\rangle)}{\sqrt{2}}$ and $|Z_-\rangle =$

$\frac{(|Z_1\rangle-|Z_2\rangle)}{\sqrt{2}}$. Analogously, symmetric $Q_+ = \frac{(Q_1+Q_2)}{\sqrt{2}}$ and antisymmetric $Q_- = \frac{(Q_1-Q_2)}{\sqrt{2}}$ coordinates are defined[10]. The Hamiltonian of the molecule expressed in terms of the $|N\rangle$,$|Z_+\rangle$ and $|Z_-\rangle$ states reads[12]

$$\begin{aligned} H_{mol} = \eta\{|Z_+\rangle\langle Z_+| + |Z_-\rangle\langle Z_-|\} + \sqrt{2}t\{|N\rangle\langle Z_+| + |Z_+\rangle\langle N|\} \\ + \hbar\omega_{vib}\left(b_+^\dagger b_+ + b_-^\dagger b_-\right)|N\rangle\langle N| \\ + \hbar\omega_{vib}\left[b_+^\dagger b_+ + b_-^\dagger b_- + \frac{\lambda_{dia}}{\sqrt{2}}\left(b_+^\dagger + b_+\right) + \lambda_{dia}{}^2\right]\{|Z_+\rangle\langle Z_+| \\ + |Z_-\rangle\langle Z_-|\} + \hbar\omega_{vib}\left[\frac{\lambda_{dia}}{\sqrt{2}}\left(b_-^\dagger + b_-\right)\right]\{|Z_+\rangle\langle Z_-| + |Z_-\rangle\langle Z_+|\} \end{aligned} \quad \text{(S5)}$$

where $b_+^\dagger$ (b_+) and $b_-^\dagger$ (b_-) are creation (annihilation) operators creating (destroying) one vibrational quantum in Q_+ and Q_-, respectively. In eq. (S5), the terms

$$\sigma = |Z_+\rangle\langle Z_+| + |Z_-\rangle\langle Z_-| \quad \text{(S6a)}$$

$$\delta = |Z_+\rangle\langle Z_-| + |Z_-\rangle\langle Z_+| \quad \text{(S6b)}$$

describe the average charge on the central donor site of the molecule, and the unbalance of the charge on the two acceptor sites of the molecule[10,11], respectively. By numerical diagonalization of the Hamiltonian in Eq. (S5) at each point in the effective coordinate plane (Q_+,Q_-), we obtain three eigenstates $|S_0\rangle$, $|S_1\rangle$ and $|S_2\rangle$ which describe the effective PESs of the A-D-A molecule in the electronic ground state (S_0) and the two first excited electronic states (S_1 and S_2). When the strengths of electronic and vibronic couplings are comparable, as in our case, $|S_1\rangle$ shows an anharmonic PES with a double-minimum along Q_-. From our experiments and supported by quantum chemical calculations, we set $\eta = 2.1$ eV, $t = 150$ meV, $\hbar\omega_{vib} = 178$ meV and $\lambda_{dia} = 0.75$. The latter corresponds to a Huang-Rhys factor of ≈ 0.3.To include the effect of a polar solvent, we use a reaction field approach[10] describing the solvent as a homogeneous polarizable medium. Following the work of Painelli and coworkers[10], the contribution of polar solvation to the effective Hamiltonian is modeled as

$$H_{sol}\,(t) = -\mu_0\, F_R(t)(|Z_+\rangle\langle Z_-| + |Z_-\rangle\langle Z_+|). \quad \text{(S7)}$$

Here μ_0 is the dipole moment and $F_R(t) = F_{R0}\left(1 - e^{-\frac{t}{\tau_{sol}}}\right)$ the time-dependent reaction field that we approximate as building up with a single exponential rise time τ_{sol}. From the experimental emission spectra, we estimate the equilibrium value $|\mu_0 F_{R0}|$ ~700 meV. By fixing

the sign of the reaction field, we assume a preferential alignment of the solvent dipoles, in contrast to the random dipole orientation in solution. We estimate $\tau_{sol} \approx 500$ fs from the build-up of ESA in DCM. This value is in good agreement with rotation correlation times deduced from the frequency-dependent dielectric function of DCM[13,14]. The effect of H_{sol} is to gradually tilt the PES of $|S_1\rangle$ along Q_- as the reaction field builds up, resulting in the solvatochromic shift in the emission[10]. The total Hamiltonian of the system including the effect of solvation is given by $H_0 = H_{mol} + H_{sol}$.

4. Quantum chemical calculations

Quantum chemical calculations are conducted to characterize the electronic and vibronic properties of the molecule within solvent environments. All calculations are performed using the Gaussian 16 package[15] employing the CAM-B3LYP/def2-SVP level of theory[16,17]. Ground state (GS) geometry optimization is carried out considering the equilibrium Polarizable Continuum Model (PCM) solvation[18]. Time-dependent density function theory (TD-DFT) calculations are further performed to evaluate electronically excited state properties. Further, the optimal geometry of the first excited state (S1) is attained through the TD-DFT calculations with equilibrium linear response (LR) solvation method[19]. Moreover, state specific solvation methods coupled with TD-DFT approach, i.e., the corrected linear-response (cLR)[20] and the external iteration (EI)[21] technique, are employed to calculate the first excited state energies of the optimized ground and excited state geometries. The orbital plots are obtained using the Multiwfn package[22,23].

4.1 Molecular geometry and permanent dipole moment. Calculated GS and S1 optimal structures in nonpolar CHX and polar DCM solvents along with the vectors of the respective state dipole moments are shown in Supplementary Table 1. In both solvents, the molecule has a planar structure on S1 state, while the GS geometries are nonplanar. Observed geometry changes from GS to S1 are similar in the two solvents. This rationalizes similar Huang-Rhys factors in the two solvents as presented below. GS geometries of the molecule in CHX and DCM have similar permanent dipole, which is parallel to the plane of the donor segment and perpendicular to the long axis of the donor part. This evidences the symmetry between the right and left sides of the quadrupolar system. For the S1 geometries, in contrast to a small dipole in CHX (~ 5 Debye), we observe formation of a significant permanent dipole in DCM (~ 15 Debye). The main component of this dipole is directed along the molecular backbone pointing from the donor (center) segment to one of the acceptor parts. This evidences the solvation induced excited state symmetry breaking. The effects of torsional disorder, present at ambient

conditions, on the dynamical symmetry breaking in polar solvents will be investigated in future studies using non-adiabatic excited state molecular dynamics methodology[24].

4.2 Excited state properties. The transition energies and oscillator strengths of the first excited state in the optimal GS and S1 geometries are summarized in Supplementary Table 2. For both GS and S1 geometries, the first excited state transition energy is redshifted in DCM compared to that in CHX. Such redshift is more pronounced in the S1 geometry (i.e., the emission point). The state-specific cLR method produces a slightly higher absorption transition energy than that of the LR method. The state-specific EI method gives the lowest excited state transition energy for a specific geometry. The oscillator strengths for all calculated transitions do not change significantly.

4.3 Orbital representation of transition densities and electron-hole distributions as well as natural transition orbitals of the S1 state calculated for the GS and S1 geometries. The polar solvent induced excited state symmetry breaking can be clearly seen from the transition densities (TDs) of the first excited state (Supplementary Fig. 13) and the natural transition orbitals (NTOs, Supplementary Fig. 14). Ground state symmetry is not affected by the solvents and the S1 wave function of the ground state geometry is symmetrically distributed over the molecule. In the nonpolar solvent CHX, the symmetry is also remained at the emission point (S1 of the first excited state geometry). In contrast, the polar solvent DCM breaks the excited state symmetry, leading to asymmetric distribution of the S1 wave function at the emission point.

4.4 Huang-Rhys factors. Based on the vibrational modes of the optimized GS and S1 geometries, the Huang-Rhys factor of mode n is evaluated as $S_n = \frac{\Delta Q_n^2}{2}$, where ΔQ_n is the dimensionless displacement along this mode inferred from the difference between the GS and S1 geometries. Such decomposition is performed with respect to the final state normal coordinates using the Dushin package[25]. In our analysis, the displacement is decomposed in terms of the S1 and GS normal modes for the absorption and emission, respectively. The dimensionless displacements along all modes and the Huang-Rhys factors are summarized in Supplementary Fig. 16.

For the molecule in both CHX and DCM solvents, three groups of modes, which are located at the low (< 100 cm^{-1}), intermediate (600 cm^{-1} ~ 710 cm^{-1}), and high (1300 cm^{-1} ~ 1600 cm^{-1}) frequency regions, exhibit detectable Huang-Rhys factors. The low frequency modes around 50 cm^{-1} are associated to the symmetric motion of the molecule backbone. The high frequency modes around 1500-1600 cm^{-1} of GS and S1 geometries are presented in Supplementary Figs.

17 and 18, respectively. For the molecule in CHX, these modes are symmetric and delocalized over the entire molecule. In contrast, in DCM, the antisymmetric modes are observed, such as the 1500.1 cm^{-1} mode of the GS geometry and the 1514.6 cm^{-1} mode of the S1 geometry which are antisymmetric in DCM.

4.5 Vibronic absorption and emission spectra. The vibronic spectra are calculated via[26]

$$I\ (\omega) = \mathrm{Im}\left[\mu_{ge}^2 \sum_{\nu_1} \cdots \sum_{\nu_{3N-6}} \frac{\prod_{n=1}^{3N-6} \langle 0|\nu_n\rangle^2}{\Omega_{ge} \pm \sum_{n=1}^{3N-6} \nu_n \omega_n - \omega - i\Gamma}\right]. \quad \text{(S8)}$$

Here, ν_n is the occupation number of mode n with a frequency of ω_n, Γ is a damping factor, Ω_{ge} and μ_{ge} are the energy and transition dipole moment magnitude for the transition between the GS and S1, respectively. Within the harmonic approximation, the Franck-Condon integrals are $\langle 0|\nu_n\rangle^2 = \frac{e^{-S_n} S_n^{\nu_n}}{\nu_n!}$, where S_n is the Huang-Rhys factor of mode n. The $+$ and $-$ sign is adopted for the simulation of the absorption and emission spectra, respectively. To facilitate the calculations, the modes in the range [100, 1750] cm^{-1} with Huang-Rhys factors larger than 0.05 (as highlighted by the dashed line in the two bottom panels of Supplementary Fig. 20) are considered. These modes and their Huang-Rhys factors are shown in the middle row of Supplementary Fig. 20. After preliminary convergence test, a cut-off value of 2 for the occupation number ν_n is adopted and can already produce converged spectra. To obtain the stick spectra, the damping factor Γ is set to be an especially small number. The transition dipole moment μ_{ge} is set to unity. The simulated stick spectra are shown in the first row of Supplementary Fig. 20 in comparison with the experimental spectra. Note that each simulated 0-0 transition stick has been shifted by an individual energy to match the highest peak of the experimental spectra.

5. Analysis of the temporal oscillations in the experimental *ΔT/T* data

The experimental identification of antisymmetric Q_- modes in pump-probe experiments is usually difficult[27,28]. For quadrupolar A-D-A dyes, no experimental studies of the effects of the antisymmetric Q_- mode on pump-probe spectra are known to us. A theoretical study by Painelli and coworkers[11], that analyzes coherent vibrational wavepacket dynamics and pump-probe spectra for a class of quadrupolar dyes undergoing symmetry breaking in the excited state, reports PES and coherent wavepacket dynamics qualitatively similar to the PES shown in Fig.

1 and dynamics shown Fig. 4 of our manuscript, respectively. In that study[11], wavepacket splitting along the Q_- coordinate causes red-shifted stimulated emission in the simulated pump-probe spectra, with an amplitude oscillating out of phase with the oscillations of the stimulated emission from the S_1 PES saddle point. The interplay of electronic and vibronic couplings, leading to the symmetry broken PES, also brightens the Q_- mode in the pump-probe spectra, suggesting that it may manifest as additional periodic oscillations superimposed to those of the Q_+ mode in the pump-probe dynamics until the wavepacket relaxes. To this end, we analyze the oscillatory modulation in the experimental $\Delta T/T$ signals in CHX and DCM that are depicted in Figs. 3c and 3d of the main manuscript, respectively.

5.1 Signatures of symmetric and antisymmetric modes in CHX. For the data in Fig 3c, Fourier transforms at each probe energy for waiting times beyond 150 fs (Supplementary Fig. 23a) show that these transients are governed by three oscillatory components, two high-frequency modes at 1457 cm^{-1} and 1406 cm^{-1} and a lower frequency one at 182 cm^{-1}. We thus fit the oscillatory modulation of the transients in Fig. 3c for time delays beyond 150 fs induced by each mode to a response function $r_i(t,\omega_d) = A_i(\omega_d)\cos(\omega_i t + \varphi_i)\exp(-t/\tau_i)$ and find decay times of $\tau_1 = 0.35$ ps, $\tau_2 = 1$ ps, and $\tau_3 = 0.30$ ps. Adding two weaker frequency components at 1520 cm^{-1} and 75 cm^{-1} help to improve the fitting of oscillations in the data (Supplementary Fig. 23b). The results are summarized in Supplementary Table 3. For the present discussion, the high-frequency oscillations at 1457 cm^{-1} and 1406 cm^{-1} are most relevant. To extract amplitude and phase of these oscillations, we keep the mode frequencies and their decay times constant and fit the transients $\Delta T/T(t,\omega_d) \cong \sum_i r_i(t,\omega_d)$ to the data in Fig. 3c for time delays beyond 150 fs. The resulting amplitudes and spectral phases for the two high-frequency modes are depicted in Supplementary Fig. 24.

The long-lived mode at 1406 cm^{-1} displays a characteristic dip in amplitude at around 2.02 eV, i.e., close to the electronic 0-0 absorption resonance. Here, the spectral phase displays a jump by roughly $\pi/2$. Both features are distinct signatures of the coupling of the electronic transition to a Franck-Condon active totally-symmetric vibration, as known for example, from the thorough analysis of the amplitude and phase profiles in femtosecond coherence spectroscopy provided by Champion and coworkers[29,30]. In the ideal case of a single-mode displaced harmonic oscillator (DHO) model, π-phase jumps appear at the 0-0 transition and near the vibronic sidebands. The phase jump near the low-energy sideband is clearly visible while the oscillation amplitude near the high-energy sideband is weak and a faithful reconstruction of the phase profile is currently difficult. For the ideal case of a single-mode DHO model, we expect

an amplitude profile that is symmetric around the 0-0 transition. The experimentally measured profile is instead red-shifted and asymmetric. Numerical simulations suggest that this amplitude profile likely arises from vibrational relaxation on the solvated PES. Periodic oscillations of the pump-probe signal also arise from cross-phase modulation due to the periodic modulation of off-resonant contributions (for example from higher-lying excited states of the molecule) to the refractive index induced by the ground-state vibrational motion. Both effects, vibrational relaxation on the solvated PES and temporal oscillations of the off-resonant contributions to the refractive index are likely to explain the differences between the measured amplitude and phase profiles and the ideal DHO model. For a recent discussion of the effects of vibrational wavepackets on pump-probe spectra, we refer to Refs [31,32]. The amplitude profile of the shorter-lived mode at 1457 cm^{-1} appears more asymmetric and shifted to lower energies. Its spectral phase profile is very similar to that measured for the 1406 cm^{-1} mode, showing again distinct phase jumps near the 0-0 transition and the lower energy vibronic sideband. The spectral-phase is shifted by π with respect to that of the 1457 cm^{-1} mode. In the DHO model, such phase shifts can arise for a finite red-shift between the excitation pulse and the 0-0 transition[29]. In our experiments, the excitation pulses are indeed slightly red-detuned from the 0-0 transition. The red-shift of the amplitude profile can again be rationalized by considering vibrational relaxation on the solvated PES and off-resonant cross-phase modulation contributions to the signals.

Also, the Raman spectrum of our A-D-A molecule (Supplementary Fig. 2) shows at least two high frequency modes around 1400 cm^{-1}, specifically 1400 cm^{-1} and 1435 cm^{-1} with lifetimes of at least 350 fs or longer. This strongly supports that the two high-frequency modes observed in the $\Delta T/T$ spectra result from totally-symmetric vibrations of the carbon backbone of the A-D-A molecule. These modes nicely account for the coherent oscillations in the experimental data in Fig. 3c for waiting times > 150 fs, as confirmed by taking the difference between the $\Delta T/T$ map and the fitted transients (Supplementary Fig. 25a). Most importantly, for waiting times < 100 fs, pronounced coherent oscillations with a period of ~ 23 fs remain. The probe energy dependence of these oscillations does not match expectations from a simple displaced harmonic oscillator, but we observe that the oscillations are in-phase across the probe energy range (Supplementary Fig. 25b). This suggests the presence of non-Condon effects[33]. We therefore take these additional, short-lived high-frequency oscillations as the signature of the antisymmetric mode in the $\Delta T/T$ spectra.

5.2 Signatures of symmetric and antisymmetric modes in DCM. Applying the same analysis to the $\Delta T/T$ signals in DCM depicted in Fig. 3d of the manuscript, we obtain two dominant high-frequency modes at 1440 cm^{-1} and 1383 cm^{-1} and lower frequency components below 200

cm^{-1}. The Fourier transform map of those signals (Supplementary Fig. 26a) is generally quite similar to that in CHX. For a quantitative analysis of the data for waiting times beyond 150 fs we take the 6 modes listed in Supplementary Table 4.

The spectral amplitude and phase profiles that are deduced for the two high-frequency modes (Supplementary Fig. 27) are very similar to those seen in CHX. In particular, very similar phase profiles are obtained, showing the similar phase jumps near the 0-0 transition and the lower energy vibronic sideband. Closer inspection reveals a slight red-shifting by ~ 100 meV as an effect of the polar solvent on the PES. The two dominant high frequency modes that persist in the residuals for the longer time delays in DCM correspond to the totally-symmetric vibrations observed both in the Raman spectra (Supplementary Fig. 2) and in the data in CHX. The slight frequency red-shift in DCM is expected. As for the data in CHX, we subtract the residuals resulting from the vibronic coupling to the long-lived symmetric vibrational modes from the data in Fig. 3d. The result of this subtraction is shown in Supplementary Fig. 28a. Again, the signal almost vanishes for waiting times beyond 150 fs confirming that the high frequency modes indeed make the dominant contribution to the long-lived oscillations. For waiting times < 100 fs, we observe again a pronounced oscillatory modulation of the signal with a period of ~23 fs. As in CHX, these short-lived oscillations appear in the region around 2.0 eV. The damping time is again of ~ 50 fs (Supplementary Fig. 28b). As in CHX, the transients cannot be simply described by the response function of totally-symmetric vibrations, but they show a more complex spectral profile.

For the short-lived oscillations shown in Supplementary Figs. 25a and 28a, the phase of the oscillations is virtually unchanged across the probe energy, in contrast to the π phase jump predicted by the displaced harmonic oscillator model. This absence of a phase jump has been reported as a distinct signature of non-Condon effects in experimental studies of excited state dynamics in stilbene[33]. We therefore assign the short-lived 23-fs period mode to an antisymmetric vibration. The rapid decay time of ~50 fs very likely reflects vibrational relaxation of the wavepacket created on the excited state potential of the A-D-A molecule.

The above experimental analysis shows that the persistent high-frequency coherent modulations of the $\Delta T/T$ dynamics in both CHX and DCM are governed by vibronic couplings to two energetically closely spaced totally-symmetric carbon backbone modes with lifetimes of 0.35 ps and 1 ps. These vibrations also dominate the Raman spectrum of the molecule. Additionally, our analysis provides strong evidence for vibronic coupling to a high-frequency antisymmetric mode, rapidly damped within less than 100 fs. Our results confirm

experimentally vibronic couplings to high-frequency symmetric Q_+ and antisymmetric Q_- modes, which are key components of phenomenological essential states models. Moreover, our results also indicate that the PES in Fig. 1 is oversimplified since it considers only one Q_+ and one Q_- mode. Instead, higher-dimensional PES[34] resulting from multimode couplings are needed for a more realistic description of the quantum dynamics.

To further support the presence of non-Condon effects, we have extracted the electronic transition dipole moment between ground and excited state from quantum chemical calculations in both solvents. These calculations (Supplementary Fig. 13) show that the total value of the transition dipole moment in our A-D-A molecule depends on the molecular geometry, which further supports the presence of non-Condon effects in both solvents.

5.3 Polarization-resolved pump-probe measurements. In general, also the analysis of the anisotropy of vibrational wavepackets in polarization-resolved pump-probe experiments may give valuable information on non-Condon effects[35,36]. We report polarization-resolved pump-probe measurements for the A-D-A molecule in CHX in Supplementary Fig. 30. For understanding the results of these measurements, it is helpful to consider first the transition dipole moments (TDM) of such an A-D-A molecule (Supplementary Fig. 29). In the general case of a symmetric A-D-A molecule with bending angle α between the two equal donor-acceptor (D-A) arms (Supplementary Fig. 29), the dipole operator, in the basis of the neutral $|N\rangle$ and zwitterionic states $|Z_1\rangle$ and $|Z_2\rangle$, reads[37]

$$\begin{aligned}\hat{\mu} &= \vec{\mu}_1|Z_1\rangle\langle Z_1| + \vec{\mu}_2|Z_2\rangle\langle Z_2| = \\ &= \mu_0\left(\sin\frac{\alpha}{2}\hat{x} + \cos\frac{\alpha}{2}\hat{z}\right)|Z_1\rangle\langle Z_1| - \mu_0\left(\sin\frac{\alpha}{2}\hat{x} - \cos\frac{\alpha}{2}\hat{z}\right)|Z_2\rangle\langle Z_2|\end{aligned} \tag{S9}$$

Here $\vec{\mu}_1$ and $\vec{\mu}_2$ are the dipole moments associated with the left and right D-A dipole, respectively (Supplementary Fig. 29, black arrows). The amplitude of the dipole μ_0 in each arm is defined by the charge and by the length of the arm, and α is the bending angle between the two D-A arms. The dipole moment has thus components along both the long (x) and short (z) axis of the molecule with values that depend on μ_0 and on the bending angle.

Coupling between the two arms of the molecule gives rise to wavefunction delocalization across the A-D-A backbone and results in three electronic eigenstates, that we denoted $|S_0\rangle$, $|S_1\rangle$, $|S_2\rangle$ in the manuscript. The resulting TDMs between the ground state S_0 and the two excited states

S_1 and S_2 are orthogonally polarized. Specifically, at the Franck-Condon point ($Q_+ = 0; Q_- = 0$), defined as the equilibrium geometry of the ground state PES, they are oriented along the long ($S_0 \rightarrow S_1$) and short ($S_0 \rightarrow S_2$) axis of the molecule, respectively. Their amplitude is governed by the bending angle[37] as

$$\vec{\mu}_{S_{01}} = \langle S_0|\hat{\mu}|S_1\rangle \propto \sin\frac{\alpha}{2}\hat{x}$$
$$\vec{\mu}_{S_{02}} = \langle S_0|\hat{\mu}|S_2\rangle \propto \cos\frac{\alpha}{2}\hat{z} \quad \text{(S10)}$$

For a bent molecule, $\alpha \neq 180°$, both orthogonally polarized transitions are optically allowed. For a quasi-linear molecule as in our case, $\alpha \approx 180°$, $\mu_{S_{02}}$ is vanishingly small and only $\mu_{S_{01}}$ is relevant for the optical spectra. Upon photoexcitation, vibronic coupling of the zwitterionic states via Q_- leads to slight oscillations of the charges on the two acceptors with a period given by Q_-, as shown in Fig. 4 of the manuscript. This gives rise to a slight modulation of the amplitude of the TDM along the long axis of the molecule, but it does not induce changes in the orientation of TDM. As such, we do not expect to obtain any specific time dependence in the anisotropy $r = \frac{T_{\parallel}-T_{\perp}}{T_{\parallel}+2T_{\perp}}$ due to impulsive excitation of the Q_- mode. Here, $T_{\parallel}$ and $T_{\perp}$ denote the intensities of the transmitted probe light for parallel and perpendicular polarization, respectively.

In our experimental data, reported in Supplementary Fig. 30, we observe, within the signal-to-noise ratio, that the differential transmission for parallel polarization is a factor of 3 larger than for perpendicular polarization, $(\Delta T/T)_{\parallel} = 3(\Delta T/T)_{\perp}$ (Supplementary Fig. 30c-f). This ratio is indeed expected from the initial anisotropy $r = \frac{T_{\parallel}-T_{\perp}}{T_{\parallel}+2T_{\perp}} = 0.4$ of an ensemble of randomly oriented linear molecules in solution[38]. Therefore, as expected from the ESM model of a linear A-D-A molecule, we indeed do not observe any clear signature of the impulsive excitation of the Q_- mode on the polarization anisotropy.

To support this conclusion, we perform numerical simulations of polarization-dependent pump-probe spectra based on the ESM model (Supplementary Fig. 31). We take the same model parameters for our linear molecule as in the manuscript (Methods and Supplementary Information section 3). We set the electronic dephasing time to $T_2 = 50$ fs and the vibrational relaxation time in the excited state to 50 fs. To account for the random orientation of the molecular dipoles in solution, we choose the linear polarization of the pump field vector such that the initial value of r is 0.4.

The simulated $\Delta T/T$ spectra for parallel and perpendicular polarization are shown in Supplementary Fig. 31 a and b, respectively. The probe wavelength range is the same as in the experiments. In agreement with the experiment, we see that the amplitude of the $\Delta T/T$ map for perpendicular polarization is reduced by a factor of 3 in comparison to that of parallel polarization. This confirms the conclusion that we cannot gain insight into the Q_- mode dynamics by analyzing the anisotropy dynamics.

Yet, there are distinct signatures of Q_- wavepacket dynamics. The most dominant signature is the periodic modulation of the amplitude of the GSB peak 2.01 eV in Supplementary Fig. 31a,b during the first ~100 fs. Such a coherent amplitude modulation would not be expected from a displaced harmonic oscillator model considering coupling to symmetric vibrational modes only. It arises from the coherent, oscillatory motion of the wavepacket along Q_-. The oscillations are damped with the vibrational relaxation time and present a clear marker for the antisymmetric mode in the pump-probe measurements. There are other, yet more subtle, signatures of the Q_- mode in these simulations. Specifically, we emphasize the amplitude beatings in the residuals in Supplementary Fig. 31c,d that are seen during the first 100 fs.

In summary, the experimental data and pump-probe simulations (Supplementary Figs. 30-31) show that the oscillations in the residuals, with a phase that is independent of the probe energy, is indeed the dominant signature of the excitation of the Q_- mode in our molecule. For such a quasi-linear molecule, the anisotropy of pump-probe measurements alone cannot reveal its excitation. This would be different for bent A-D-A molecules with non-vanishing TDMs pointing along the long and short axis of the molecule.

6. Time-resolved fluorescence of the A-D-A molecule in CHX and DCM

To further quantify the amount of symmetry breaking, we have now performed time-resolved fluorescence measurements of our A-D-A molecule in nonpolar CHX and polar DCM. The time resolution of the experiment is ~ 100 ps. The data are shown below in Supplementary Fig. 32. In CHX (Supplementary Fig. 32a), solvation is complete within the time resolution of our measurement, resulting in an emission spectrum that closely resembles the static PL spectrum in Fig. 2a of the manuscript. The PL decays with a time constant of ~ 1.34 ns (Supplementary Fig. 32c, black). The measured PL spectrum is only slightly red-shifted with respect to the SE spectrum labeled $S_{1,sol}$ (red curve in Fig. 2e of the manuscript), which is extracted as the most long-lived emission component from the global analysis of our pump-probe data. Thus, solvation is already mostly completed after 1 ps in CHX. In DCM (Supplementary Fig. 32b), the transient PL spectrum is strongly red-shifted to around 1.45 eV. The spectral shape agrees

well with the static spectrum reported in Fig. 2b of the manuscript. Slight color shifts arise from different quantum yields of the detectors in the two measurements. Also in DCM, the shape of the transient PL is essentially time-independent, implying the solvation is complete within our time resolution. The PL decay in DCM is shortened to 0.27 ns. In DCM, the PL spectrum again has a similar lineshape as the slowest SE component in the global analysis, $S_{1,sol}$ (black curve in Fig. 2f of the manuscript). Here, $S_{1,sol}$ is centered around 1.7 eV, in contrast to the fully solvated PL spectrum at 1.45 eV in Supplementary Fig. 32b. Therefore, in DCM, solvation is not yet complete after 1 ps and additional slower relaxation towards the fully solvated spectrum is expected.

7. Compound characterization data

The synthetic steps are reported in the methods section. Nuclear magnetic resonance (NMR) is recorded on a Bruker DRX 400 using residual solvents signals as internal standard. ^{1}H-NMR (400 MHz, $CDCl_3$, refer to Supplementary Figure 3 for the chemical structure): δ [ppm] = 7.94-7.88 (m, 4H, H6,9), 7.81(s, 2H, H4‘‘), 7.75-7.69 (m, 6H, H5,7,8), 7.28 (s, 2H, H4‘), 6.99 (s, 2H, H3), 4.03 (d, ^{3}J=5.46 Hz, 2H, Hα), 2.82 (s, 8H, Hλ,λ‘), 2.03-2.01 (m, 1H, Hβ), 1.78-1.69 (m, 8H, Hμ,μ‘), 1.50-1.22 (m, 48H, Hγ-ι,γ‘-η‘,ν-ο,ν‘-ο‘), 0.95-0.90 (m, 12H, Hπ,π‘), 0.87-0.82 (m, 6H, Hκ,θ‘). Due to low solubility, ^{13}C-NMR measurements could not be conducted. High resolution mass spectra (HR-MS) are recorded using the Fourier-Transform Ion Cyclotron Resonance (FTICR) mass spectrometry: $[M]^+$ calculated for $C_{84}H_{101}NO_4S_6$: *m/z* = 1379.60548; found: *m/z* = 1379.60398 (*δm/m*= 1.09 ppm). The NMR and mass spectra are reported in Supplementary Figs. 3 and 4, respectively.

Supplementary Tables

Supplementary Table 1. Geometries and permanent dipole moments of the GS and S1 states in their respective optimized geometries in CHX and DCM solvents.

solvent	dipole information	GS geometry	S1 geometry
CHX	vector (Debye)	(-1.74, 2.96, -2.99)	(-1.08, 1. 90, -1.85)
	magnitude (Debye)	4.55	2.87
	Geometry	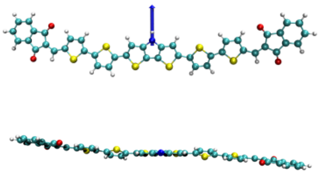	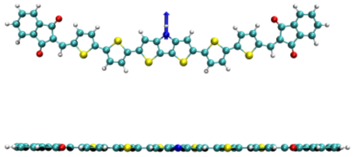
DCM	vector (Debye)	(-1.98, 3.37, -3.34)	(-14.51, -4.74, -0.69)
	magnitude (Debye)	5.14	15.29
	Geometry	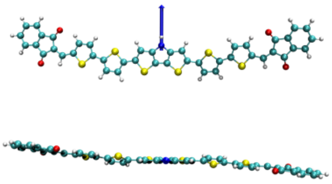	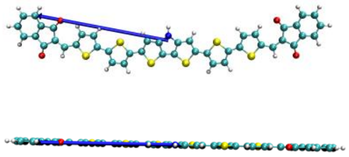

Supplementary Table 2. First excited transition energies and their respective oscillator strength (in parentheses) of the ground state and first excited state geometries in CHX and DCM solvents

solvent	method	GS geometry (absorption), eV	S1 geometry (emission), eV
CHX	LR	2.52 (3.95)	1.99 (4.05)
	cLR	2.56 (3.95)	2.07 (4.05)
	EI	2.49 (3.71)	2.02 (3.91)
DCM	LR	2.48 (3.94)	1.89 (3.77)
	cLR	2.51 (3.94)	1.93 (3.70)
	EI	2.42 (3.69)	1.78 (3.64)

Supplementary Table 3. Dominant vibrational modes and their decay times τ for the A-D-A molecule in CHX retrieved from the analysis of the pump-probe data in Fig. 3c of the manuscript for waiting times beyond 150 fs.

**Mode #**	**Frequency (cm^{-1})**	**Decay time (ps)**
1	1457	0.35
2	1406	1.0
3	182	0.3
4	75	> 10
5	1520	1.0

Supplementary Table 4. Dominant vibrational modes and their decay times τ in DCM retrieved from the analysis of the data in Fig. 3d of the manuscript for waiting times beyond 150 fs.

**Mode #**	**Frequency (cm^{-1})**	**Decay time (ps)**
1	1440	0.35
2	1373	1.0
3	117	0.3
4	1491	1.0
5	59	> 10
6	713	2.0

Supplementary Figures

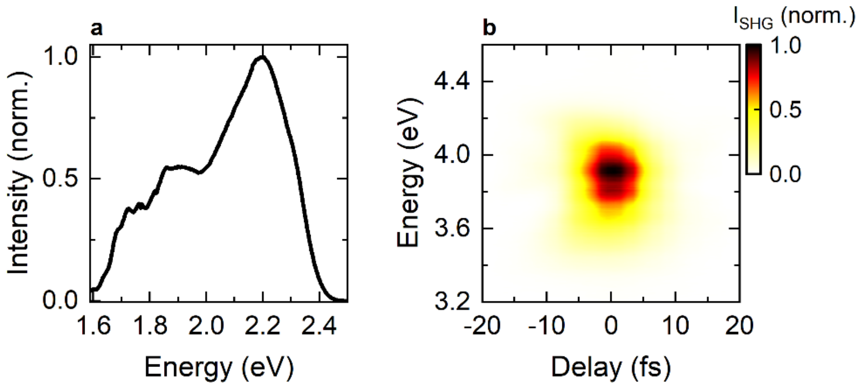

Supplementary Fig. 1. a, Normalized laser spectrum of the NOPA pulses. **b,** SHG-FROG of the cross-correlation between the pump and probe beams. The cross-correlation trace shows a full width at half maximum (FWHM) of 11 fs. This points to FWHM of the intensity profile of the pump and probe pulses of ~ 8 fs.

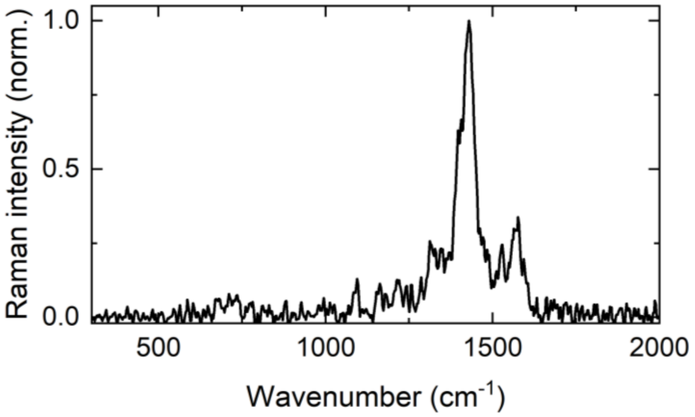

Supplementary Fig. 2. Raman spectrum of the A-D-A molecule in powder form.

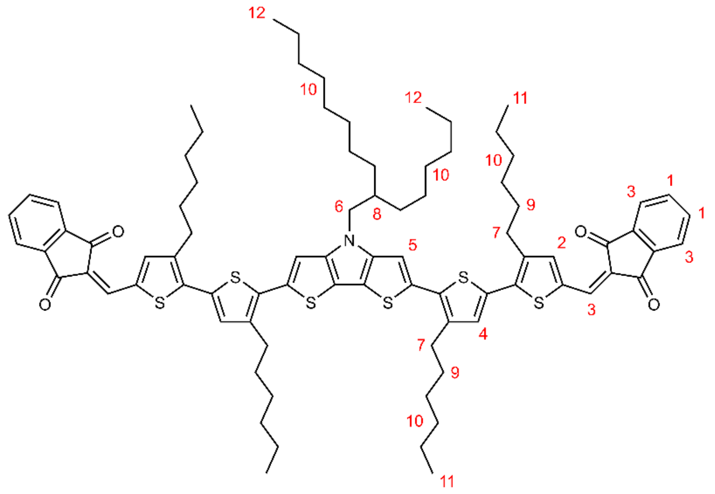

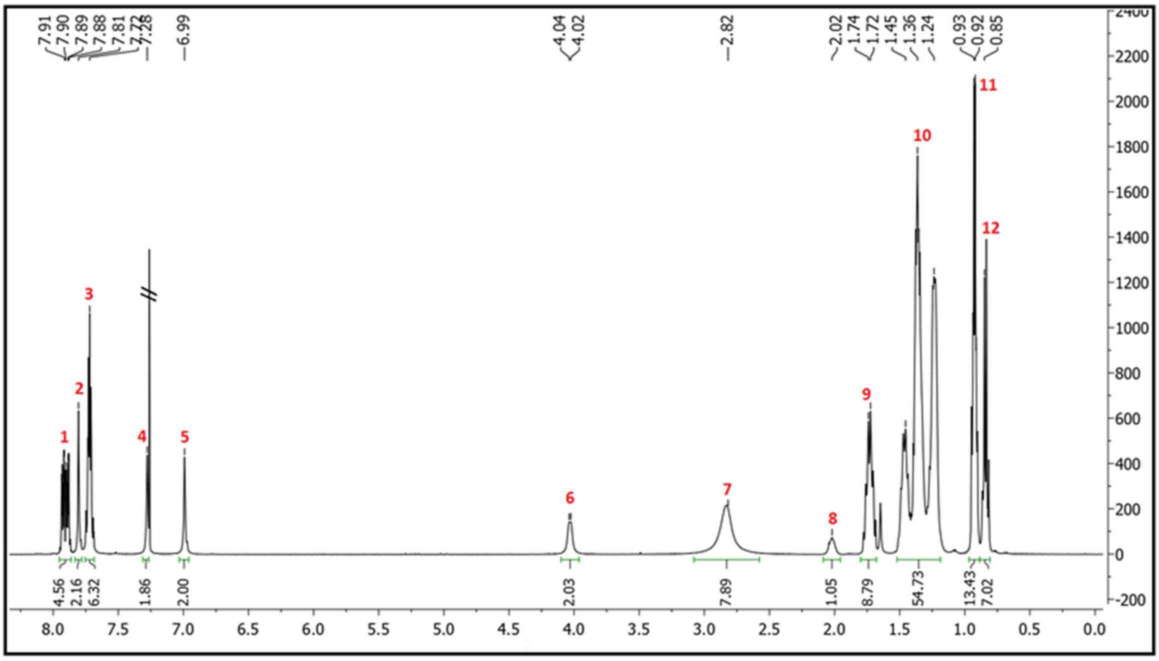

Supplementary Fig. 3. Chemical structure of the molecule with assignment of protons (top) and ^{1}H-NMR spectrum in $CDCl_3$ (bottom).

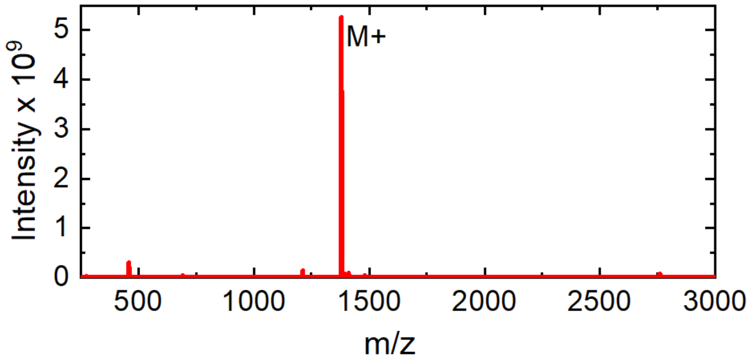

Supplementary Fig. 4. High resolution mass spectrum (HRMS-FTICR) of the ADA molecule. [M]+ calculated for $C_{84}H_{101}NO_4S_6$: m/z = 1379.60548; found: m/z = 1379.60398 (δm/m= 1.09 ppm).

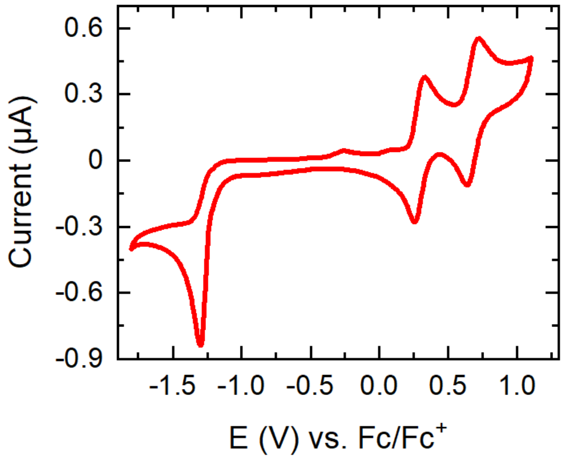

Supplementary Fig. 5. Cyclic voltammogram of the A-D-A molecule (indicated as **3** in the Extended data figure 1 in the main manuscript) in dichloromethane/tetrabutylammonium hexafluorophosphate (0.1 M), scan speed 100 mV s^{-1} at room temperature referenced against Fc/Fc^{+}.

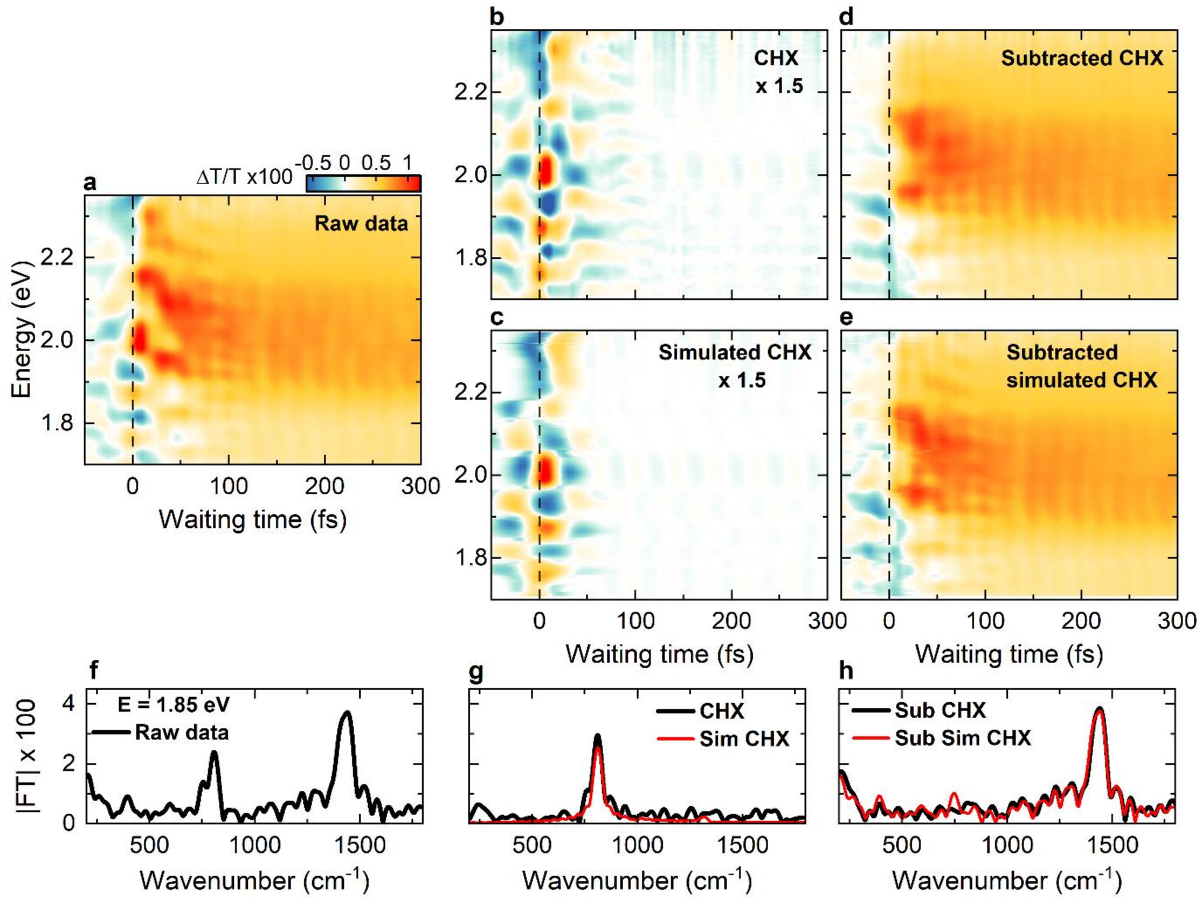

Supplementary Fig. 6. Solvent subtraction from the $\Delta T/T$ spectra for the nonpolar solvent. **a,** Raw differential transmission $\Delta T/T$ map of the A-D-A molecules in CHX solution. **b**, Reference measurement of neat CHX solvent recorded under identical experimental conditions as in a. The data show cross-phase modulation (XPM) around time zero and vibrational wavepacket motion in the electronic ground state of the solvent induced by impulsive stimulated Raman scattering (ISRS). **c**, Simulations of the solvent response based on Eqs. S1 and S2. **d,e**, Solvent-corrected $\Delta T/T$ maps obtained after subtracting (d) the reference measurement in b or (e) the simulated solvent response in c from the raw data in a. **f-h**, Fourier transforms of the pump-probe measurement for waiting times beyond 110 fs at selected probe energy of $E_D = 1.85$ eV performed on the raw data in a (f), the experimental

and simulated neat solvent data in b and c (g) and the data after solvent subtraction in d and e (h). The FT map of the A-D-A data after solvent subtraction in h is dominated by the high-frequency carbon-carbon stretching modes of the A-D-A molecules at 1430 cm^{-1}.

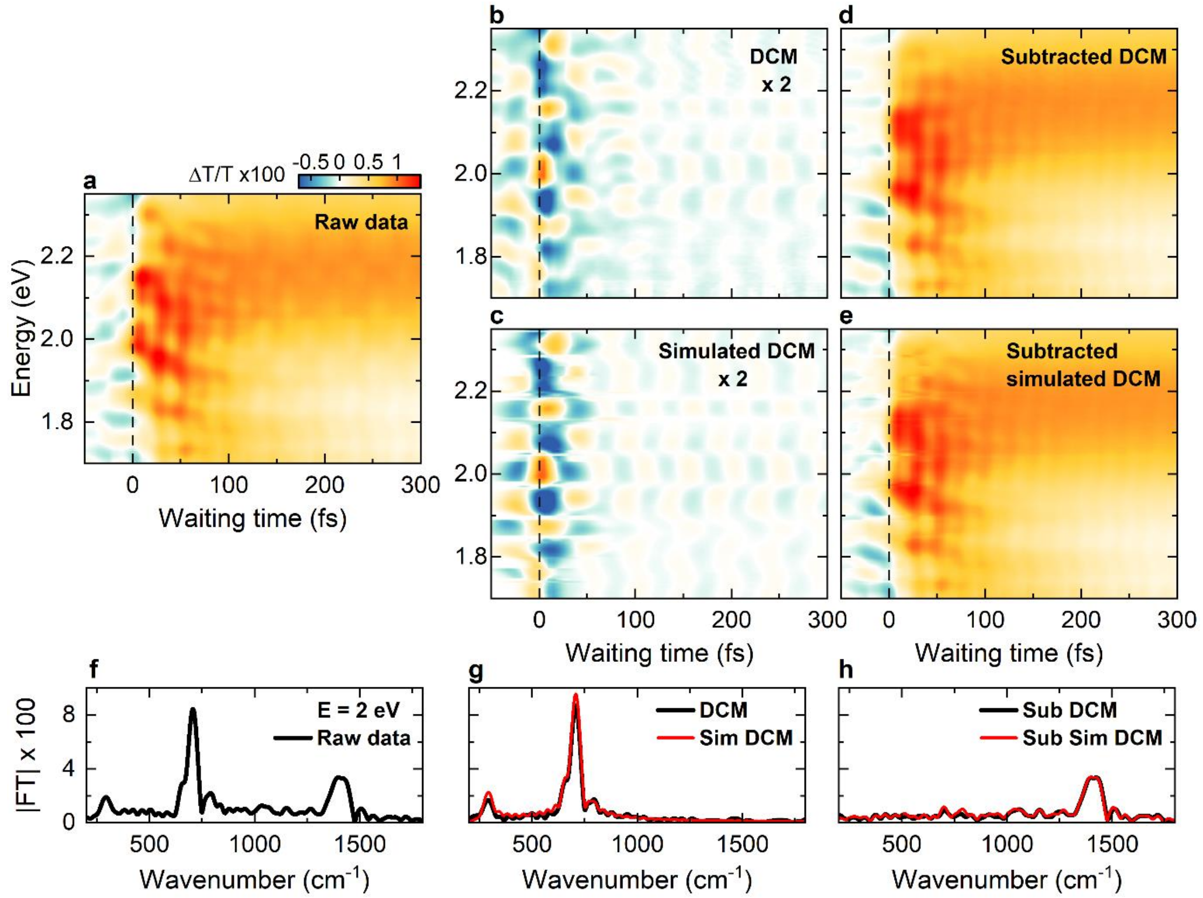

Supplementary Fig. 7. Solvent subtraction for the polar solvent. **a**, Raw differential transmission $\Delta T/T$ map of the A-D-A molecules in DCM solution. **b**, Reference measurement of neat DCM solvent recorded under identical experimental conditions as in a. The data show XPM around time zero and ground-state vibrational wavepacket motion induced by ISRS. **c**, Simulations of the solvent response based on Eqs. S1 and S2. **d,e**, Solvent-corrected $\Delta T/T$ maps obtained after subtracting (d) the reference measurement in b or (e) the simulated solvent response in c from the raw data in a. **f-h**, Fourier transforms of the pump-probe measurements for waiting times beyond 110 fs at selected probe energy of $E_D = 2.0$ eV performed on the raw data in a (f), the experimental and simulated neat solvent data in b and c (g) and the data after solvent subtraction in d and e (h). The FT map of the A-D-A data after solvent subtraction in h is dominated by the high-frequency carbon-carbon stretching modes of the A-D-A molecules at 1430 cm^{-1}.

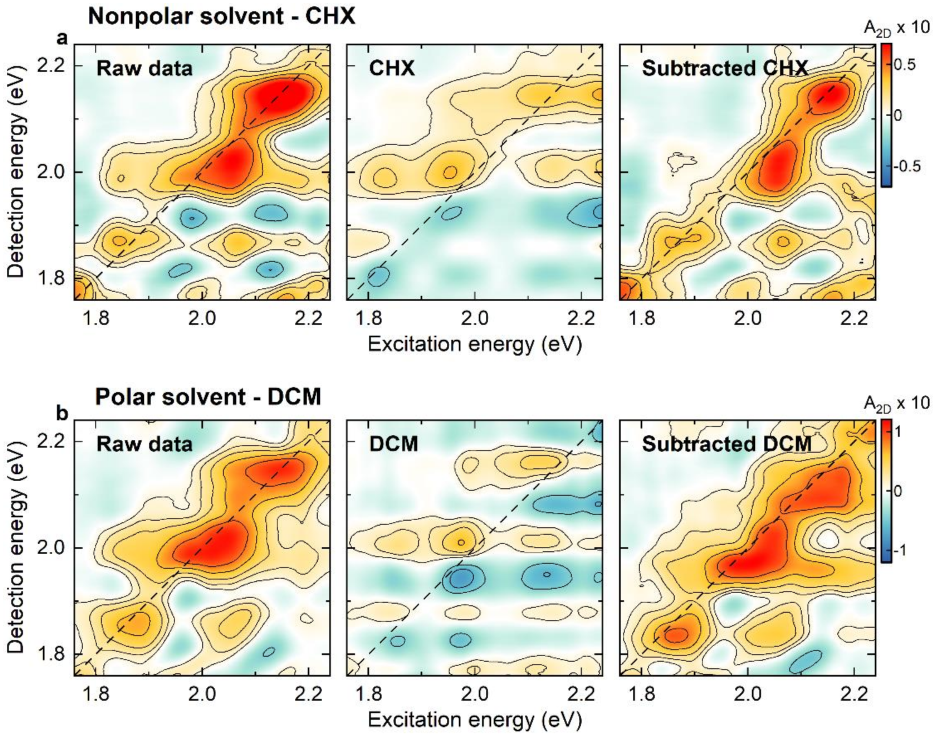

Supplementary Fig. 8. Subtraction of the solvent response from the 2DES maps for an exemplary early waiting time of 10 fs.

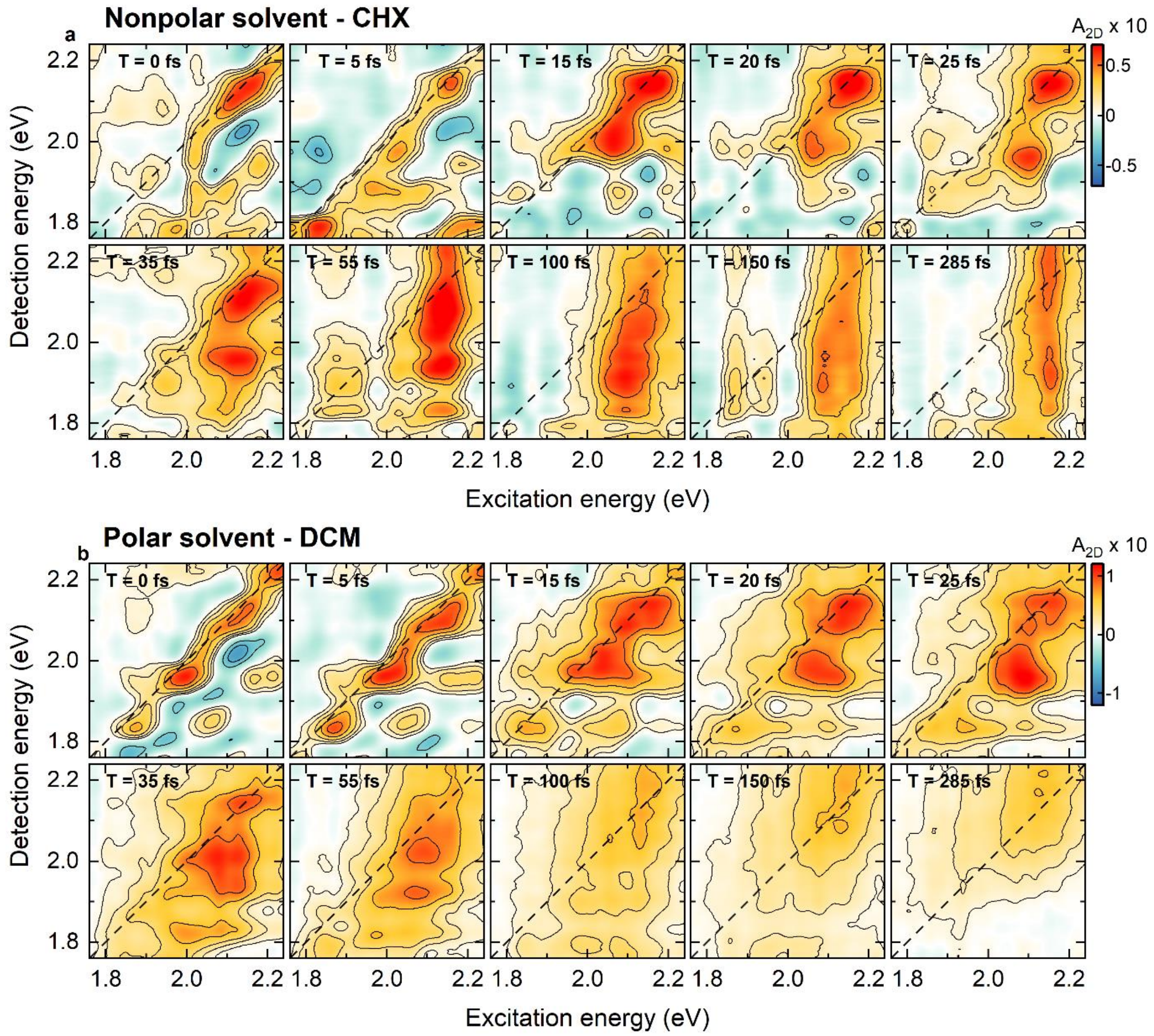

Supplementary Fig. 9. Absorptive 2DES maps of the A-D-A molecule in (a) nonpolar and (b) polar solvents at selected waiting times.

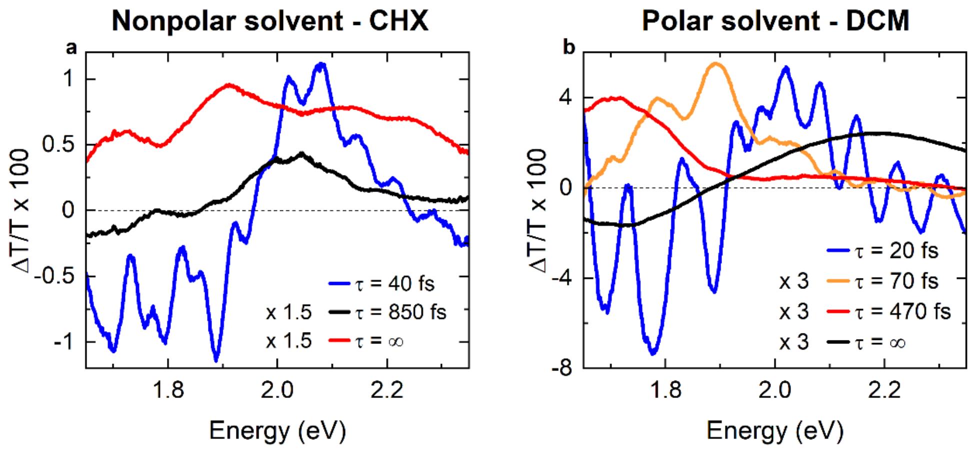

Supplementary Fig. 10. DADS spectra and their associated decay times resulting from a global analysis of the differential transmission maps of A-D-A molecule dissolved in (a) CHX and (b) DCM.

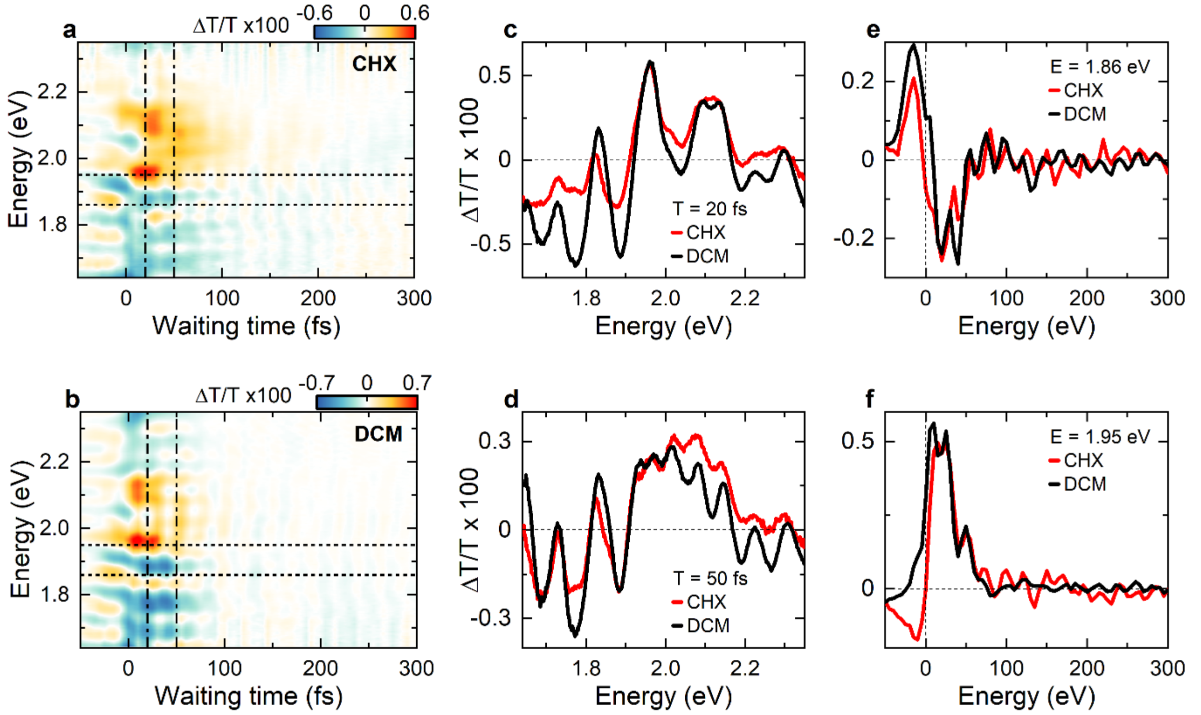

Supplementary Fig. 11. a,b, Spectral residuals of the differential transmission maps at early times in (a) CHX and (b) DCM obtained by subtracting the long-lived GSB, SE and ESA spectra from the corresponding experimental maps. In DCM (b), additionally, also the short-lived, vibronically structured SE spectrum (Fig. 2f, green) has been subtracted. **c,d,** Spectra at selected waiting times of (c) T = 20 fs and (d) T = 50 fs, marked by vertical dashed-dotted lines in a,b. **e,f,** Dynamics at selected detection energies of (e) E_D = 1.86 eV and (f) E_D = 1.95 eV, marked by horizontal dotted lines in a,b.

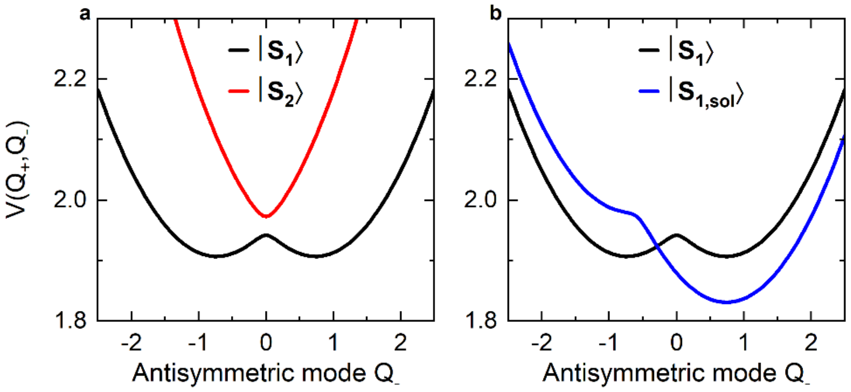

Supplementary Fig. 12. a, Schematic potential energy surfaces (PESs) of the excited states obtained from the essential state model in the absence of the solvent. The PESs are plotted as a function of Q_- at a fixed value of $Q_+ = 0.75$, corresponding to the minimum of $|S_1\rangle$ along Q_+ showing the characteristic double-minimum potential in $|S_1\rangle$. b, PES of the first excited state along Q_- at a fixed value of $Q_+ =$ 0.75 as in (a) in the absence ($|S_1\rangle$, black) and presence ($|S_{1,sol}\rangle$, green) of the interaction with the polar DCM solvent.

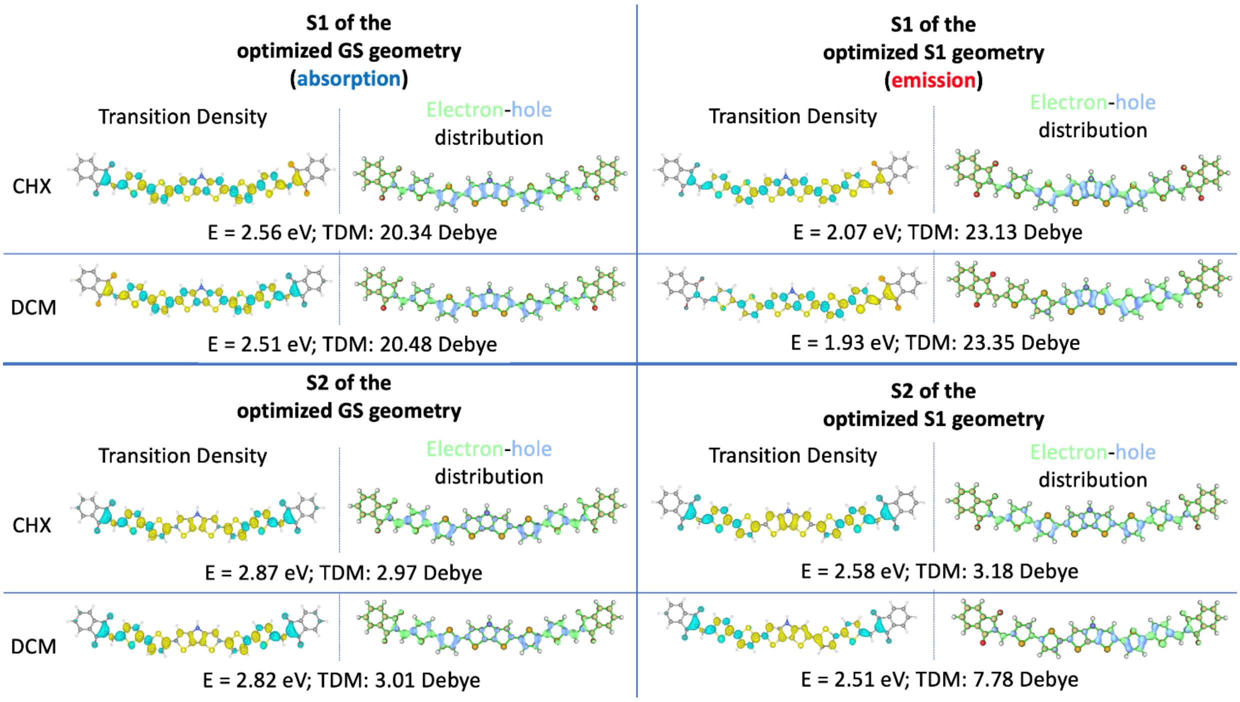

Supplementary Fig. 13. Orbital representations of the transition density (isosurface value=0.001) and electron-hole distributions (isosurface value=0.002) of (upper row) the S1 and (lower row) S2 states evaluated for the optimal GS and S1 structures. Shown are the results from calculations using the cLR method. The transition energies and transition dipole moments (TDMs) for the two geometries are also shown. The latter suggest the presence of non-Condon effects.

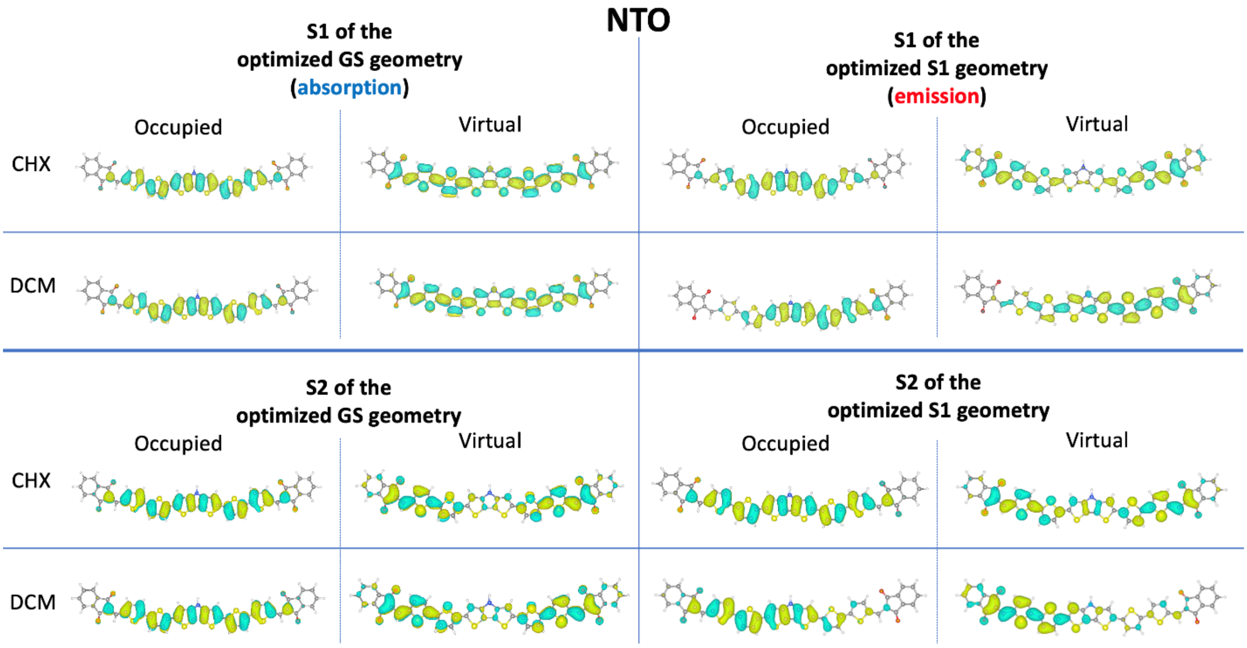

Supplementary Fig. 14. Natural transition orbitals (NTOs) (isosurface value=0.02) of (upper row) the S1 and (lower row) S2 states calculated for the optimal GS and S1 structures. Results for the S1 geometry are from calculations using the cLR method.

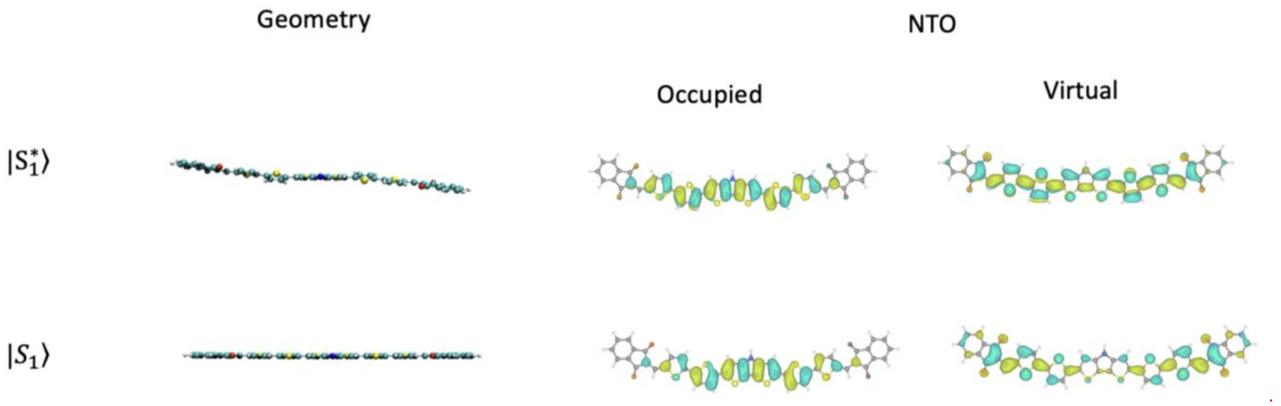

Supplementary Fig. 15. Geometry and natural transition orbitals (NTOs) for the S_1* and S_1 states.

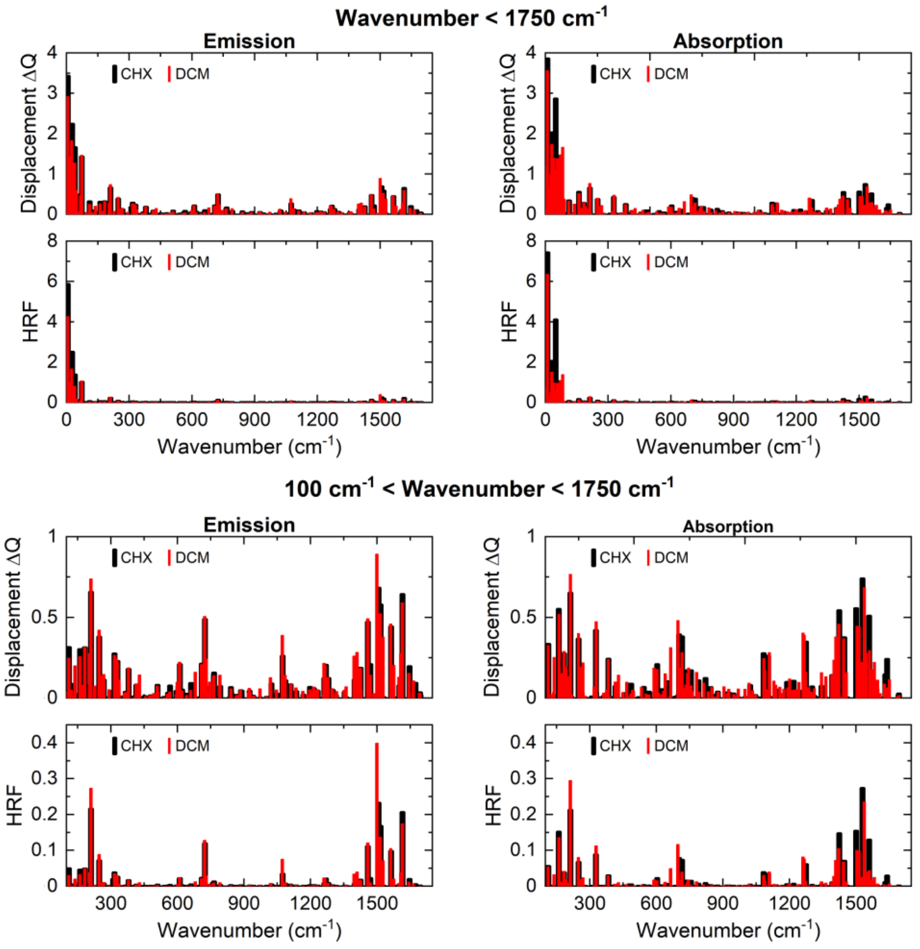

Supplementary Fig. 16. Calculated Huang-Rhys factors and dimensionless displacements for molecule in DCM and CHX.

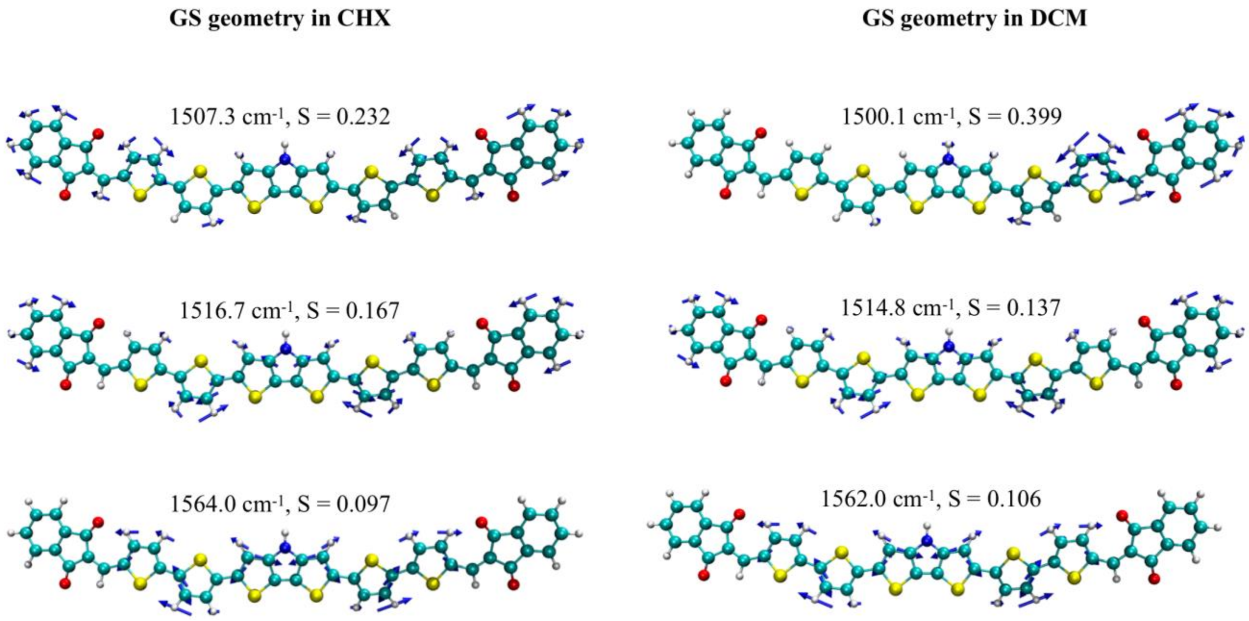

Supplementary Fig. 17. Vibrational modes near 1500 cm^{-1} frequency range evaluated for the GS geometry in CHX (left) and DCM (right).

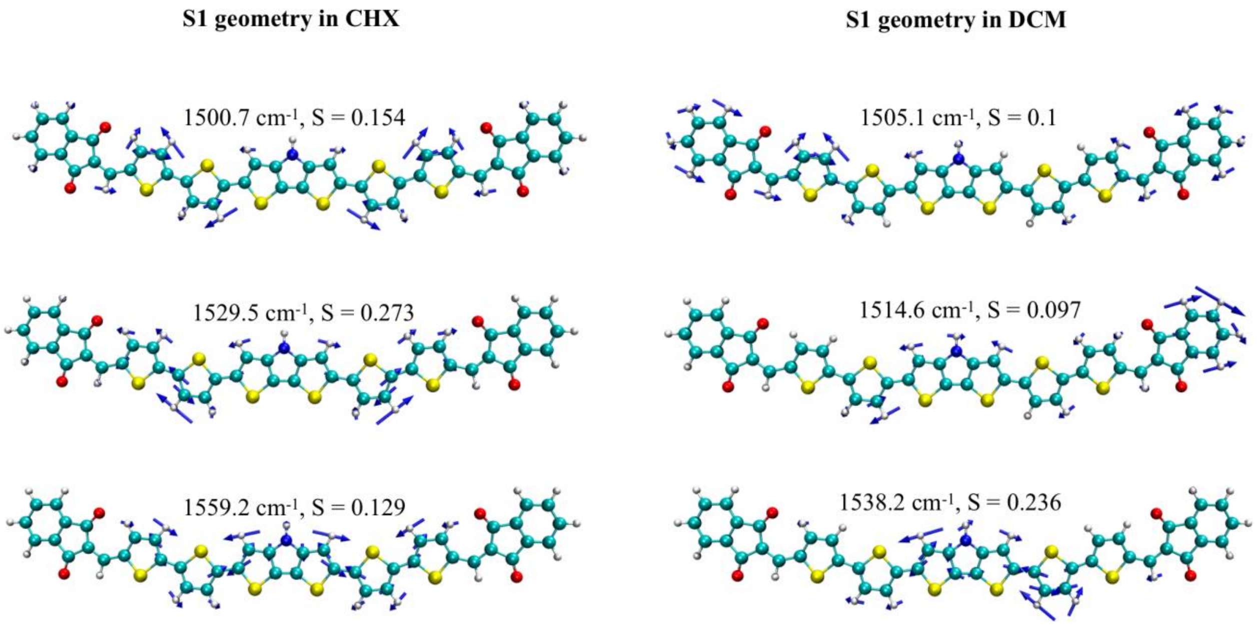

Supplementary Fig. 18. Vibrational modes near 1500 cm^{-1} range evaluated for the S1 geometry in CHX (left) and DCM (right).

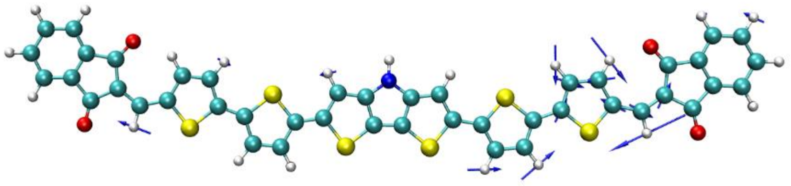

Supplementary Fig. 19. Vibrational mode at 1432 cm^{-1} of the optimal $S_{1,Sol}$ geometry in DCM. This is a "broken symmetry" structure in polar solvent, where the direction of vibrational motion modifies the bond-length pattern on one side of the molecule and contributes to the localization of the electronic excitation in this region. As such, this mode is a good candidate representing the Q_1 mode.

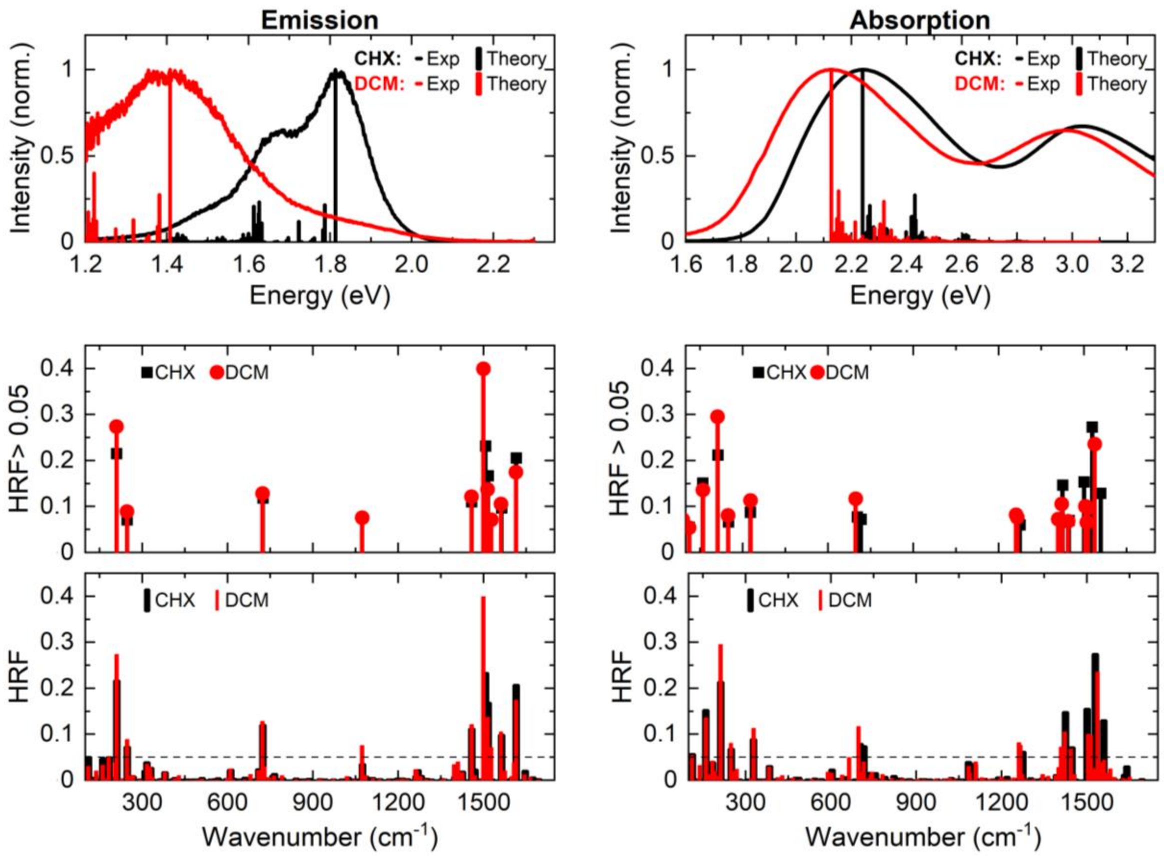

Supplementary Fig. 20. Vibronic emission (left) and absorption (right) spectra of the A-D-A molecule in DCM (red) and CHX (black). Solid curves are the measured spectra and the calculated spectra are presented by the sticks. Each simulated 0-0 transition stick has been shifted by an individual energy to match the highest peak of the experimental spectra. The Huang-Rhys factors used in spectra simulations are presented in the middle row.

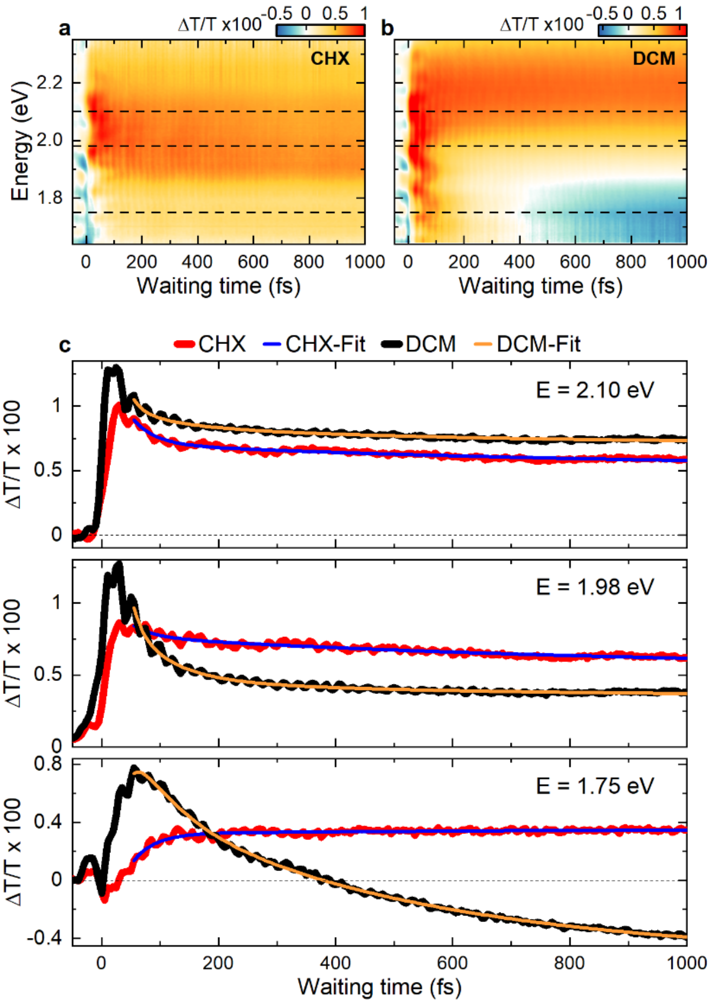

Supplementary Fig. 21. Differential transmission ΔT/T maps of the quadrupolar molecule in (**a**) CHX and (**b**) DCM together with (**c**) cross-sections showing the dynamics at selected probe energies around the ground state bleaching (2.01 eV), stimulated emission (1.98 eV) and stimulated emission/excited state absorption (1.75 eV). Fits to the data obtained using the decay times extracted from the global analysis are shown overlapped on each dynamic.

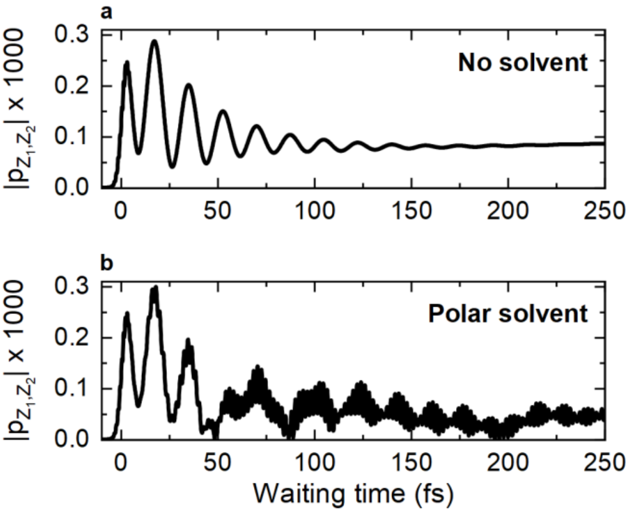

Supplementary Fig. 22. Electronic coherence between the local zwitterionic states $|Z_1\rangle$ and $|Z_2\rangle$ in the absence and presence of polar solvent.

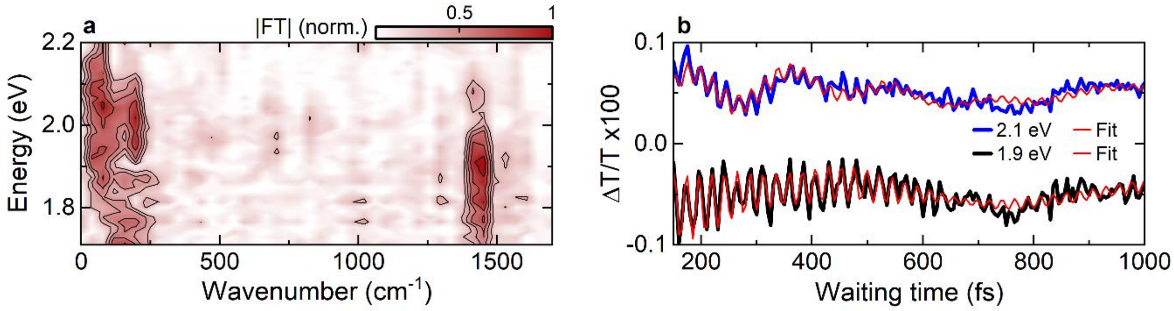

Supplementary Fig. 23. a, Amplitude of the Fourier transform of the residual differential transmission map for the A-D-A molecule in CHX obtained from the data in Fig. 3c of the manuscript for waiting times beyond 150 fs. **b,** Time-domain residual oscillations at two exemplary probe energies together with the corresponding fits.

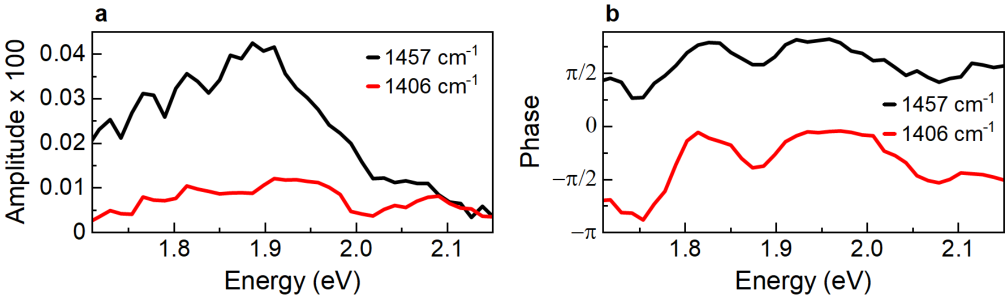

Supplementary Fig. 24. a, Amplitude and **b,** spectral phase of the two high frequency modes at 1457 cm-1 (with a decay time of $\tau_1 = 0.35$ ps) and 1406 cm-1 ($\tau_2 = 1$ ps) in CHX as a function of the detection energy.

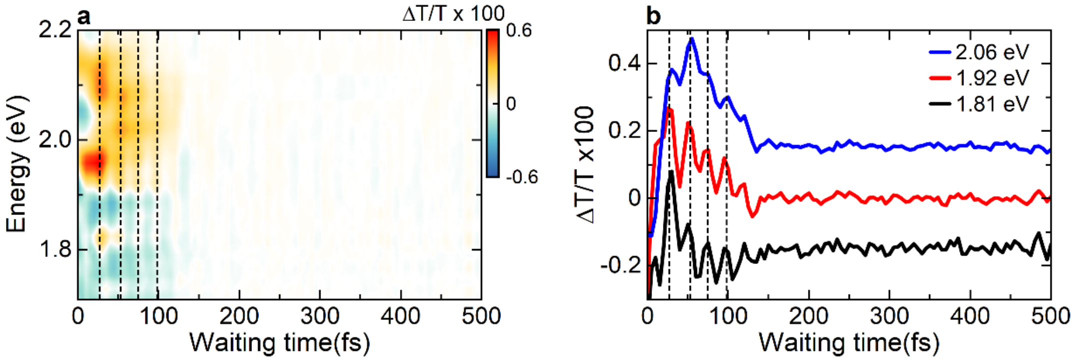

Supplementary Fig. 25. a, Difference between the pump-probe map in CHX in Fig. 3c and the coherent oscillations induced by the totally-symmetric vibrational modes in Supplementary Table 3. Pronounced high-frequency oscillations with a period of ~ 23 fs are seen for waiting times below 100 fs in the region around the 0-0 transition. These are the signatures of antisymmetric vibrational modes in the pump-probe spectra. **b**, Cross-sections at selected probe energies.

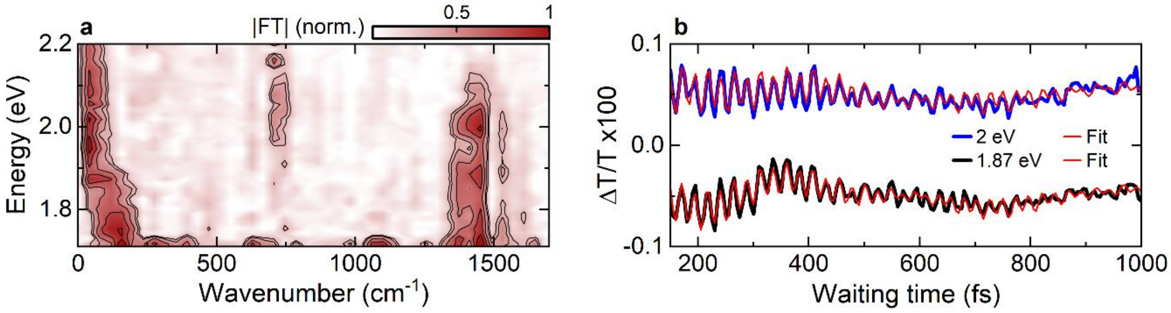

Supplementary Fig. 26. a, Amplitude of the Fourier transform of the data in Fig. 3d of the manuscript calculated beyond 150 fs. **b,** Exemplary time-domain residual oscillations for waiting times beyond 150 fs along with the respective fits.

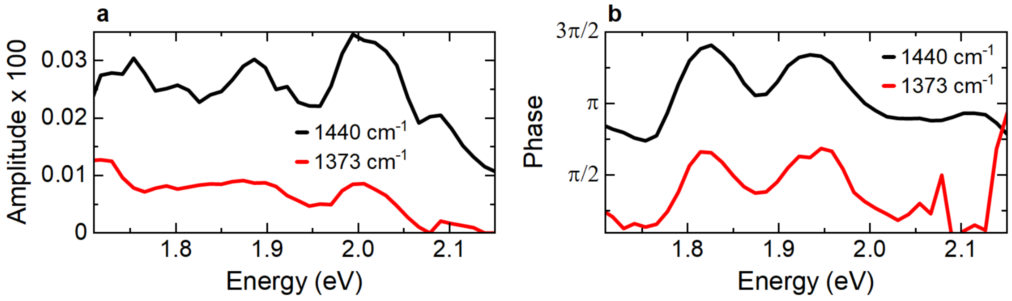

Supplementary Fig. 27. a, Amplitude and **b,** spectral phase of the two high frequency modes at 1440 cm^{-1} (with a decay time of τ_1 =0.35 ps) and 1373 cm^{-1} (τ_2 =1 ps) in DCM as a function of detection energy.

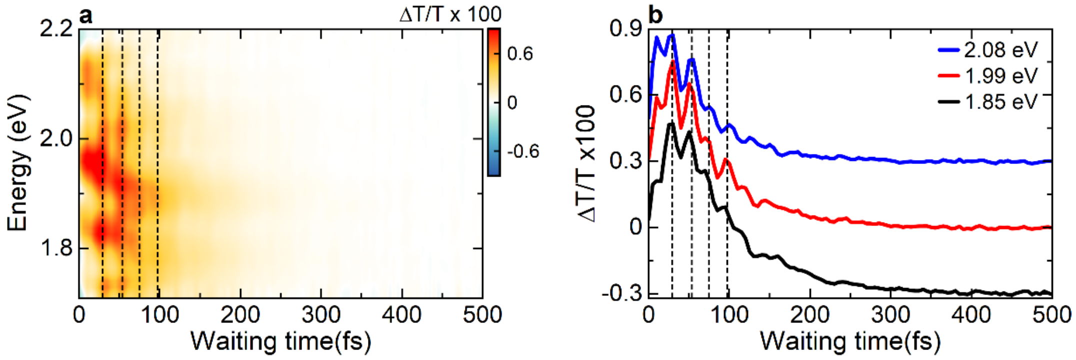

Supplementary Fig. 28. a, Difference between the pump-probe map in DCM in Fig. 3d of the manuscript and the coherent oscillations induced by the totally-symmetric vibrational modes reported in Supplementary Table 4. Pronounced high-frequency oscillations with a period of ~ 23 fs are seen for waiting times below 100 fs in the region around the 0-0 transition. These are the signatures of antisymmetric vibrational modes in the pump-probe spectra. **b**, Cross-sections at selected probe energies.

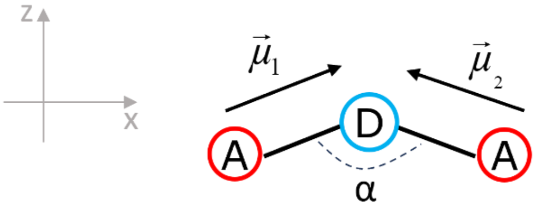

Supplementary Fig. 29. Scheme of a symmetric A-D-A molecule with an arbitrary bending angle α between the two equal D-A arms. The dipoles $\vec{\mu}_1$ and $\vec{\mu}_2$ on each arm of the molecule are directed from the negative (A) to the positive (D) charge (black arrows).

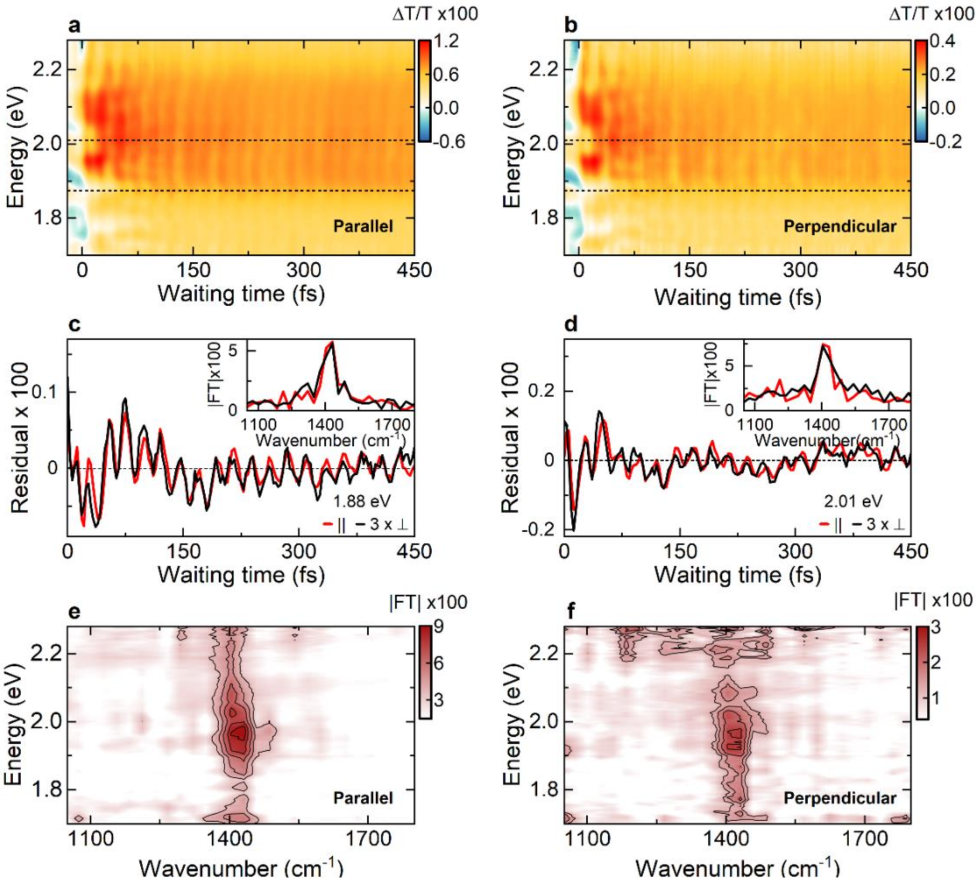

Supplementary Fig. 30. a,b, Differential transmission $\Delta T/T$ maps of the A-D-A molecule in CHX for (a) parallel and (b) perpendicular polarization between pump and probe pulses. Dashed lines indicate two exemplary probe energies for GSB at 2.01 eV and SE at 1.88 eV. **c,d,** Exemplary time-domain residual oscillations at the two selected probe energies indicated in a,b. The corresponding Fourier transform amplitudes are shown in the insets. The data for perpendicular polarization (black) are multiplied by a factor of 3. **e,f,** Fourier transform amplitude maps of the residual oscillations extracted from the $\Delta T/T$ maps in (a,b) as a function of the probe energy. All measurements show an amplitude ratio of 3 between parallel and perpendicular polarization, $(\Delta T/T)_{\parallel} = 3(\Delta T/T)_{\perp}$. This matches the anisotropy that is expected for an isotropic ensemble of linear molecules.

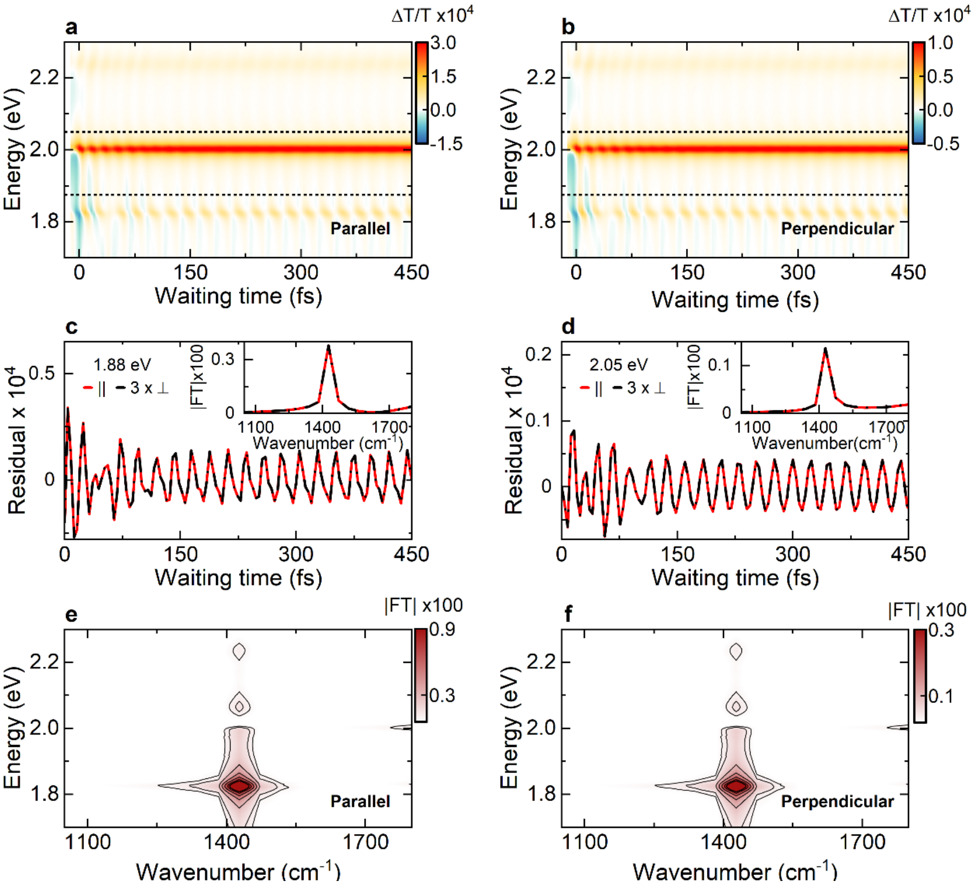

Supplementary Fig. 31. a,b, Simulated differential transmission $\Delta T/T$ maps for our A-D-A molecule for (a) parallel and (b) perpendicular polarization between pump and probe fields. The simulations are performed using the ESM model with the same parameters as reported in section 3 above and in the manuscript. The electronic dephasing time is $T_2 = 50$ fs, longer than the vibrational period (23 fs). The vibrational relaxation time in the excited state is taken as 50 fs. **c,d,** Exemplary time-domain residual oscillations at the two selected probe energies around (c) the SE peak at 1.88 eV and (d) the GSB peak at 2.05 eV. The probe energies are indicated as dashed lines in (a,b). The corresponding Fourier transforms are shown in the insets. The data for perpendicular polarization (black) between pump and probe are multiplied by a factor of 3. **e,f,** Fourier transform amplitude maps of the residual oscillations extracted from the $\Delta T/T$ maps in (a,b) as a function of probe energy and wavenumber. All data show an amplitude ratio of 3 between parallel and perpendicular polarizations. This matches the anisotropy seen in the experimental data in Supplementary Fig. 30. Importantly, the GSB band at 2.01 eV shows a clear amplitude modulation with a period of ~23 fs during the first 100 fs. This is the signature of the impulsive excitation of the Q_- mode.

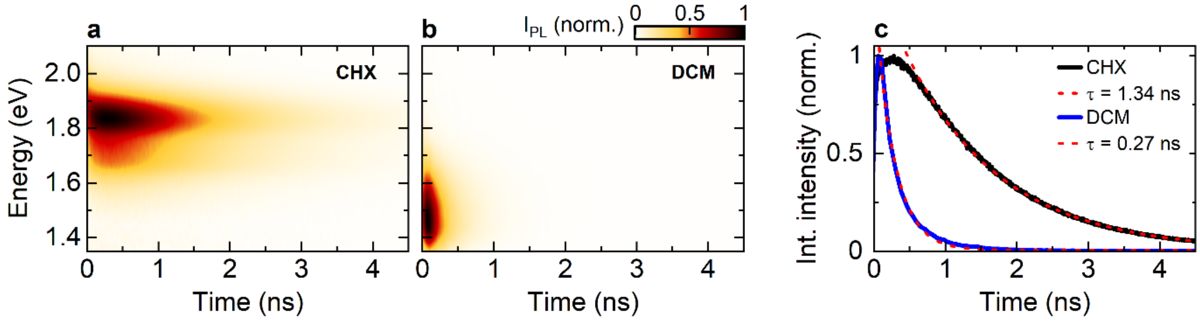

Supplementary Fig. 32. a,b, Time-resolved fluorescence spectra of the A-D-A molecule dissolved in (a) CHX and (b) DCM. **c,** Fluorescence decay dynamics integrated over the entire spectral range in (a,b) for (black) CHX and (blue) DCM. The red dashed curves are exponential fits to the data. The resulting fluorescence decay time is of 1.34 ns for CHX and 0.27 ns for DCM.

References

1 Bresci, A. *et al.* Removal of cross-phase modulation artifacts in ultrafast pump–probe dynamics by deep learning. *APL Photonics* **6**, 076104 (2021).

2 Baudisch, B. *Time Resolved Broadband Spectroscopy from UV to NIR - Beneficial Use of the Coherent Artifact and Pyrene Dynamics* PhD thesis, LMU München, (2017).

3 Ruhman, S., Joly, A. G. & Nelson, K. A. Coherent molecular vibrational motion observed in the time domain through impulsive stimulated Raman-scattering. *Ieee Journal of Quantum Electronics* **24**, 460-469 (1988).

4 Liebel, M., Schnedermann, C., Wende, T. & Kukura, P. Principles and Applications of Broadband Impulsive Vibrational Spectroscopy. *J Phys Chem A* **119**, 9506-9517 (2015).

5 Monacelli, L. *et al.* Manipulating Impulsive Stimulated Raman Spectroscopy with a Chirped Probe Pulse. *J Phys Chem Lett* **8**, 966-974 (2017).

6 Garcia, M. V. & Redondo, M. I. Identification of the stable conformer of cyclohexane by vibrational spectroscopy. *Journal of Chemical Education* **62**, 887 (1985).

7 Brother, F. E., Palma, S. M. & Sathianandan, K. 58-64 (Springer US).

8 van Stokkum, I. H., Larsen, D. S. & van Grondelle, R. Global and target analysis of time-resolved spectra. *Biochim Biophys Acta* **1657**, 82-104 (2004).

9 Rabe, M. Spectram: A MATLAB® and GNU Octave Toolbox for Transition Model Guided Deconvolution of Dynamic Spectroscopic Data. *Journal of Open Research Software* **8**, (2020).

10 Terenziani, F., Painelli, A., Katan, C., Charlot, M. & Blanchard-Desce, M. Charge Instability in Quadrupolar Chromophores: Symmetry Breaking and Solvatochromism. *Journal of the American Chemical Society* **128**, 15742-15755 (2006).

11 Sissa, C., Delchiaro, F., Di Maiolo, F., Terenziani, F. & Painelli, A. Vibrational coherences in charge-transfer dyes: a non-adiabatic picture. *J Chem Phys* **141**, 164317 (2014).

12 Zheng, C., Zhong, C., Collison, C. J. & Spano, F. C. Non-Kasha Behavior in Quadrupolar Dye Aggregates: The Red-Shifted H-Aggregate. *The Journal of Physical Chemistry C* **123**, 3203-3215 (2019).

13 Hunger, J., Stoppa, A., Thoman, A., Walther, M. & Buchner, R. Broadband dielectric response of dichloromethane. *Chemical Physics Letters* **471**, 85-91 (2009).

14 Maroncelli, M., MacInnis, J. & Fleming, G. R. Polar Solvent Dynamics and Electron-Transfer Reactions. *Science* **243**, 1674-1681 (1989).

15 Gaussian16. *Gaussian Inc.* (2019).

16 Yanai, T., Tew, D. P. & Handy, N. C. A new hybrid exchange–correlation functional using the Coulomb-attenuating method (CAM-B3LYP). *Chemical Physics Letters* **393**, 51-57 (2004).

17 Weigend, F. & Ahlrichs, R. Balanced basis sets of split valence, triple zeta valence and quadruple zeta valence quality for H to Rn: Design and assessment of accuracy. *Physical Chemistry Chemical Physics* **7**, 3297-3305 (2005).

18 Miertuš, S., Scrocco, E. & Tomasi, J. Electrostatic interaction of a solute with a continuum. A direct utilizaion of AB initio molecular potentials for the prevision of solvent effects. *Chemical Physics* **55**, 117-129 (1981).

19 Scalmani, G. *et al.* Geometries and properties of excited states in the gas phase and in solution: Theory and application of a time-dependent density functional theory polarizable continuum model. *The Journal of Chemical Physics* **124**, 094107 (2006).

20 Caricato, M. *et al.* Formation and relaxation of excited states in solution: A new time dependent polarizable continuum model based on time dependent density functional theory. *The Journal of Chemical Physics* **124**, 124520 (2006).

21 Improta, R., Barone, V., Scalmani, G. & Frisch, M. J. A state-specific polarizable continuum model time dependent density functional theory method for excited state calculations in solution. *The Journal of Chemical Physics* **125**, 054103 (2006).

22 Lu, T. & Chen, F. Multiwfn: A multifunctional wavefunction analyzer. *Journal of Computational Chemistry* **33**, 580-592 (2012).

23 Liu, Z., Lu, T. & Chen, Q. An sp-hybridized all-carboatomic ring, cyclo[18]carbon: Bonding character, electron delocalization, and aromaticity. *Carbon* **165**, 468-475 (2020).

24 Nelson, T. R. *et al.* Non-adiabatic Excited-State Molecular Dynamics: Theory and Applications for Modeling Photophysics in Extended Molecular Materials. *Chemical Reviews* **120**, 2215-2287 (2020).

25 Reimers, J. R. A practical method for the use of curvilinear coordinates in calculations of normal-mode-projected displacements and Duschinsky rotation matrices for large molecules. *The Journal of Chemical Physics* **115**, 9103-9109 (2001).

26 Karabunarliev, S., Baumgarten, M., Bittner, E. R. & Müllen, K. Rigorous Franck–Condon absorption and emission spectra of conjugated oligomers from quantum chemistry. *The Journal of Chemical Physics* **113**, 11372-11381 (2000).

27 Albrecht, A. C. On the Theory of Raman Intensities. *The Journal of Chemical Physics* **34**, 1476-1484 (1961).

28 Liebel, M., Schnedermann, C. & Kukura, P. Vibrationally Coherent Crossing and Coupling of Electronic States during Internal Conversion in $\ensuremath{\beta}$-Carotene. *Physical Review Letters* **112**, 198302 (2014).

29 Kumar, A. T. N., Rosca, F., Widom, A. & Champion, P. M. Investigations of amplitude and phase excitation profiles in femtosecond coherence spectroscopy. *The Journal of Chemical Physics* **114**, 701-724 (2001).

30 Kumar, A. T. N., Rosca, F., Widom, A. & Champion, P. M. Investigations of ultrafast nuclear response induced by resonant and nonresonant laser pulses. *The Journal of Chemical Physics* **114**, 6795-6815 (2001).

31 Rather, S. R., Fu, B., Kudisch, B. & Scholes, G. D. Interplay of vibrational wavepackets during an ultrafast electron transfer reaction. *Nature Chemistry* **13**, 70-76 (2021).

32 Rather, S. R., Weingartz, N. P., Kromer, S., Castellano, F. N. & Chen, L. X. Spin–vibronic coherence drives singlet–triplet conversion. *Nature* **620**, 776-781 (2023).

33 Ishii, K., Takeuchi, S. & Tahara, T. Pronounced Non-Condon Effect as the Origin of the Quantum Beat Observed in the Time-Resolved Absorption Signal from Excited-State cis-Stilbene. *The Journal of Physical Chemistry A* **112**, 2219-2227 (2008).

34 Picconi, D. Quantum dynamics of the photoinduced charge separation in a symmetric donor–acceptor–donor triad: The role of vibronic couplings, symmetry and temperature. *The Journal of Chemical Physics* **156**, 184105 (2022).

35 Kim, J., Lee, H. S. & Kim, C. H. Observation of Coherent Symmetry-Breaking Vibration by Polarization-Dependent Femtosecond Spectroscopy. *The Journal of Physical Chemistry B* **128**, 1053-1060 (2024).

36 Farrow, D. A., Smith, E. R., Qian, W. & Jonas, D. M. The polarization anisotropy of vibrational quantum beats in resonant pump-probe experiments: Diagrammatic calculations for square symmetric molecules. *The Journal of Chemical Physics* **129**, 174509 (2008).

37 Zhong, C., Bialas, D., Collison, C. J. & Spano, F. C. Davydov Splitting in Squaraine Dimers. *The Journal of Physical Chemistry C* **123**, 18734-18745 (2019).

38 O'Connor, D. V. & Phillips, D. *Time-correlated single photon counting*, Academic Press (1984).
